# Distributed synthesis of sarcolemmal and sarcoplasmic reticulum membrane proteins in cardiac myocytes

**DOI:** 10.1007/s00395-021-00895-3

**Published:** 2021-10-28

**Authors:** Vladimir Bogdanov, Andrew M. Soltisz, Nicolae Moise, Galina Sakuta, Benjamin Hernandez Orengo, Paul M. L. Janssen, Seth H. Weinberg, Jonathan P. Davis, Rengasayee Veeraraghavan, Sandor Györke

**Affiliations:** 1 The Frick Center for Heart Failure and Arrhythmia, College of Medicine, Dorothy M. Davis Heart and Lung Research Institute, The Ohio State University Wexner Medical Center, Columbus, OH USA; 2 Department of Physiology and Cell Biology, College of Medicine, The Ohio State University, Columbus, OH USA; 3 Department of Biomedical Engineering, College of Engineering, The Ohio State University, Columbus, OH USA

**Keywords:** Heart, Translation, Local protein synthesis, Membrane proteins

## Abstract

It is widely assumed that synthesis of membrane proteins, particularly in the heart, follows the classical secretory pathway with mRNA translation occurring in perinuclear regions followed by protein trafficking to sites of deployment. However, this view is based on studies conducted in less-specialized cells, and has not been experimentally addressed in cardiac myocytes. Therefore, we undertook direct experimental investigation of protein synthesis in cardiac tissue and isolated myocytes using single-molecule visualization techniques and a novel proximity-ligated in situ hybridization approach for visualizing ribosome-associated mRNA molecules for a specific protein species, indicative of translation sites. We identify here, for the first time, that the molecular machinery for membrane protein synthesis occurs throughout the cardiac myocyte, and enables distributed synthesis of membrane proteins within sub-cellular niches where the synthesized protein functions using local mRNA pools trafficked, in part, by microtubules. We also observed cell-wide distribution of membrane protein mRNA in myocardial tissue from both non-failing and hypertrophied (failing) human hearts, demonstrating an evolutionarily conserved distributed mechanism from mouse to human. Our results identify previously unanticipated aspects of local control of cardiac myocyte biology and highlight local protein synthesis in cardiac myocytes as an important potential determinant of the heart’s biology in health and disease.

**Supplementary Information:**

The online version contains supplementary material available at 10.1007/s00395-021-00895-3.

## Introduction

Membrane proteins, such as ion channels, transporters, and receptors, are crucial determinants of cellular biology and physiology. This is especially true in electrically excitable cells, such as cardiac myocytes, where they play key roles in the generation and propagation of action potentials, and in contractility. Despite significant progress in studying the structure and function of cardiomyocyte membrane transport proteins [9, 23, 32, 35, 54], fundamental aspects of their biology, vis-à-vis synthesis, processing, and trafficking remain poorly understood. In this regard, classical cell biology holds to a central dichotomy between cytosolic proteins and membrane proteins: cytosolic proteins are synthesized throughout the cellular space by translating mRNAs delivered through intracellular trafficking on ribosomes found in the cytoplasm. Indeed, cardiac contractile proteins (cytosolic) have been recently shown to be synthesized in the cytoplasmic space, utilizing mRNA trafficked from the perinuclear region along microtubules [33, 48]. In contrast, membrane proteins are thought to follow the classical secretory pathway for their synthesis. It is generally thought that messenger RNA (mRNA) for membrane proteins, such as ion channels, are more restricted and only centrally translated in the perinuclear rough endoplasmic reticulum (ER) and newly synthesized proteins are processed in the Golgi network before being trafficked from perinuclear regions to sites of deployment throughout the cell [25]. Previous studies investigating the trafficking of some cardiac membrane proteins, such as connexin43 (Cx43; encoded by *Gja1*), supported this view [49–51]. However, the principle that all membrane proteins follow the secretory pathways does not hold true in all mammalian cell types. Indeed, in highly polarized, structurally heterogeneous cells, such as neurons and glial cells, membrane proteins can be synthesized locally, near their sites of deployment, conferring major advantages in terms of the cell’s adaptability and efficiency [24, 26, 30, 38].

Cardiac myocytes are large, polarized, terminally differentiated cells (100 µm long × 20 µm wide × 10 µm deep) organized into periodic contractile modules called sarcomeres. Much of the ER in a cardiac myocyte, termed the sarcoplasmic reticulum (SR), is distributed throughout the myocyte, is specialized to store large amounts of Ca^2+^, and wraps itself around the contractile apparatus. SR Ca^2+^ is rapidly released and re-sequestered during each heartbeat through the successive engagement of ryanodine receptor Ca^2+^ release channels (RyR2; encoded by *Ryr2*) and SR Ca^2+^ ATPase (SERCA2a; encoded by *Atp2a2*), respectively. To mediate excitation–contraction (EC) coupling, the SR closely abuts (< 20 nm) invaginations of the sarcolemma, transverse tubules (T-tubules), that carry voltage dependent Ca^2+^ channels. Local control of SR Ca^2+^ release is a well-established hallmark of cardiac myocyte Ca^2+^ signaling [10]. Under both physiological and pathological stress, cardiac myocytes undergo hypertrophic growth, driven in significant part by new protein synthesis, including membrane proteins, and new sarcomere assembly, along with new SR and T-tubule elements [19, 48, 53]. However, the mechanisms underlying such protein synthesis in the normal and hypertrophic heart remain unclear. Therefore, we examined where and how membrane proteins are produced in adult mouse cardiac myocytes. We utilized a combined experimental and computational modeling approach, which included single-molecule mRNA visualization and a novel proximity-ligated in situ hybridization approach for selectively visualizing ribosome-associated mRNA molecules of a specific protein species. We identify here, for the first time, that the molecular machinery for membrane protein synthesis and processing occurs throughout the cardiac myocyte, and enables distributed synthesis of membrane proteins within sub-cellular niches using on-site mRNA provided, at least in part, by microtubule trafficking. We also identify distributed localization of membrane protein mRNA in both healthy and failing (hypertrophied) human ventricular tissue, demonstrating an evolutionarily conserved distributed mechanism from mouse to human. Taken together, our results identify previously unanticipated aspects of cardiac myocyte biology and highlight distributed synthesis of membrane proteins in cardiac myocytes as an important potential determinant of the heart’s adaptability in health and disease.

## Methods

All animal procedures were approved by Institutional Animal Care and Use Committee at The Ohio State University and performed in accordance with the Guide for the Care and Use of Laboratory Animals published by the U.S. National Institutes of Health (NIH Publication No. 85–23, revised 2011). A brief description of the experimental and simulation methods is provided below with more detailed descriptions included in the Supplement.

### Adult mouse cardiac tissue sections

Mouse hearts were isolated and frozen in optimal cutting temperature medium for cryosectioning as previously described [39, 52]. Cryosections (5 µm thick) were used for RNA labeling experiments.

### Adult mouse cardiac myocyte isolation and primary culture

Cardiac myocytes were enzymatically isolated from 8- to 12-week-old male C57BL6 mice and maintained in primary culture using the methods described by Ackers-Johnson et al. [1].

### Pharmacological perturbations

Myocytes were treated with the following pharmacological agents:

i) Colchicine, microtubule inhibitor [(Sigma-Aldrich, Cat #: C9754), applied at 10 µM for 8 and 24 h]
ii) Cytochalasin-D, acto-myosin inhibitor [(Sigma-Aldrich, Cat #: C8273), applied at 10 µM for 8 and 24 h]
iii) Puromycin, translation inhibitor [(Sigma-Aldrich, Cat #: P9620), applied at 200 µg/ml for 1.5 h]
iv) Cycloheximide, translation–elongation inhibitor [(Sigma-Aldrich, Cat #: 01,810), applied at 50 µg/ml for 1.5 h]
v) MHY1485, mTOR activator [(Sigma-Aldrich, Cat #: 5,005,540,001), applied at 2 µM for 3 h].

### Fluorescent immunolabeling

Tissue sections and cells were fixed using paraformaldehyde and immunolabeled as previously described [5, 52]. Briefly, samples were permeabilized, treated with blocking agent, and labeled with primary antibodies (overnight at 4 °C). To label components of the protein synthesis machinery, we used well-established antibodies, the specificity of which was verified by either genetic methods or immunogold staining [34, 37, 45, 55, 58, 61]. Target selectivity was further corroborated by independent antibody testing using two different antibodies with non-overlapping epitopes for the same protein target. The list of antibodies includes: Sec61b (Abcam, ab15576) [61], Sec61a1 (ThermoFisher Scientific, PA1-21,773), Rpl22 (Novus, NBP1-06,069) [34], Sec23a (Novus Biologicals, NBP2-34,842 and ThermoFisher Scientific, PA1-069A) [22, 60], TGN38 (Bio-Rad Laboratories, AHP499G and ThermoFisher Scientific, PA1-84,496) [13, 55, 58], and GM130 (BD, 558,712 and 610,823) [37, 45]. Subsequently, samples were labeled with fluorescent secondary antibodies before being mounted in ProLong Gold.

### *RNAscope *in situ* hybridization*

Individual mRNA molecules were visualized using RNAScope Multiplex Fluorescent Reagent Kit v2, implemented per manufacturer-recommended protocols. Briefly, isolated cells and fresh-frozen tissue sections were fixed using paraformaldehyde, dehydrated using ethanol, and stored in 100% ethanol at -20 °C. Samples were then rehydrated for hybridization and incubation steps performed per manufacturer’s recommendations.

### *Messenger RNA–ribosomal RNA proximity-ligated *in situ* hybridization (MR-PLISH)*

To visualize sites of active translation for a specific mRNA species, we developed MR-PLISH. Our method builds on a method previously published by Lewis and colleagues [33], increasing specificity and improving compatibility with various mRNA species. Briefly, probes hybridized to the mRNA species of interest and 18S ribosomal RNA (*Rn18s*) were conjugated, respectively, to digoxigenin (DIG) and 2,4-dinitrophenol (DNP). These were in turn labeled with anti-DIG and anti-DNP antibodies and a proximity ligation approach used to generate fluorophores in situ through a rolling circle polymerization reaction at sites where anti-DIG and anti-DNP antibodies occur within ~ 40 nm of each other [6, 59].

### Confocal microscopy and image analysis

Samples were imaged using an A1R-HD laser scanning confocal microscope with individual fluorophores being imaged sequentially. Additionally, in a subset of cases, a differential interference contrast (DIC) image was collected concurrently via a transmitted light detector. Image analysis was then performed using morphological object localization (MOL), a custom algorithm implemented in Matlab (Mathworks Inc, Natick, MA), to assess signal abundance as well as localization (distance from fluorescent signals to closest point on the nuclear perimeter).

### Statistics

Signal localization data presented as mean cumulative distribution functions (CDFs) with shaded regions around CDFs indicating standard deviations (SD). To ensure robustness of this approach, we also compared mean CDFs ± SD with median CDFs ± median absolute deviation (MAD) and global CDFs (calculated by pooling distance measurements from all cells within each group). All three approaches yielded CDFs that were in close agreement (Supplementary Fig. 1). Pair-wise differences between mean CDFs were evaluated using the two-sample Kolmogorov–Smirnov test. Signal density is presented as the median ± MAD, and pair-wise differences were evaluated using the two-sample Wilcoxon rank sum test. An α value of 0.05 was used for all statistical tests. The number of experiments is indicated in the figure legends. All data are presented as median ± MAD unless otherwise noted.

### Mathematical modeling

Using a well-established approach [12, 14], we simulated the distribution of mRNA in the cytoplasm using a simple trafficking model, the 1D advection–diffusion partial differential equation $\frac{\partial {u}_{i}}{\partial t}-D\frac{{\partial }^{2}{u}_{i}}{\partial x}+{a}_{i}\frac{\partial {u}_{i}}{\partial x}=-d\cdot {u}_{i},$ where ${u}_{i}$ is the concentration of the *i* type mRNA. The equation is defined on the non-dimensional spatial domain [0,1], for which x = 0 and 1 represents the edge of the nucleus and the cell membrane, respectively. At boundary x = 0, an influx of mRNA ${F}_{i}$ represents the flux of mRNA into the cytoplasm, and at boundary x = 1, we assume no-flow conditions. Experimental data for the steady-state spatial distribution of mRNA, as a function of the distance from the nucleus, were used to fit the diffusion coefficient *D*, the flow ${F}_{i}$, and the uniform velocity ${a}_{i}$, using nonlinear least squares curve fitting. Degradation rate $d$ is assumed to be the same for all mRNA species, and velocity ${a}_{i}$ is set to 0 for colchicine conditions.

### Human heart samples

Human heart tissue research was approved by The Ohio State University Institutional Review Board in accordance with all relevant ethical regulations. Informed consent for tissue collection for research use was obtained from transplant patients and families of donors. Human heart samples used in this study were freshly frozen blocks of tissue deidentified and labeled with 6-digit random reference codes. Failing heart samples were obtained from patients with left-ventricular hypertrophy and non-ischemic heart failure, while non-failing control samples were from healthy donors with no history of heart failure. Samples were obtained from The Ohio State University Cardiac Research Tissue Program and in collaboration with the LifeLine of Ohio Organ Procurement Organization. All hearts obtained were immediately flushed with cardioplegic solution following removal from donors/patients as described previously [11]. The hearts were transferred to the laboratory (within 10–30 min) in cold cardioplegic solution containing (in mM): 110 NaCl, 16 KCL, 16 MgCl_2_, 10 NaHCO_3_, and 0.5 CaCl_2_, and tissue blocks were frozen in optimal cutting temperature medium for cryosectioning as described above.

## Results

### Distinct distribution of sarcolemmal (SL) and sarcoplasmic reticulum (SR) membrane protein mRNA in murine myocardium and isolated cardiac myocytes

**Fig. 1:**
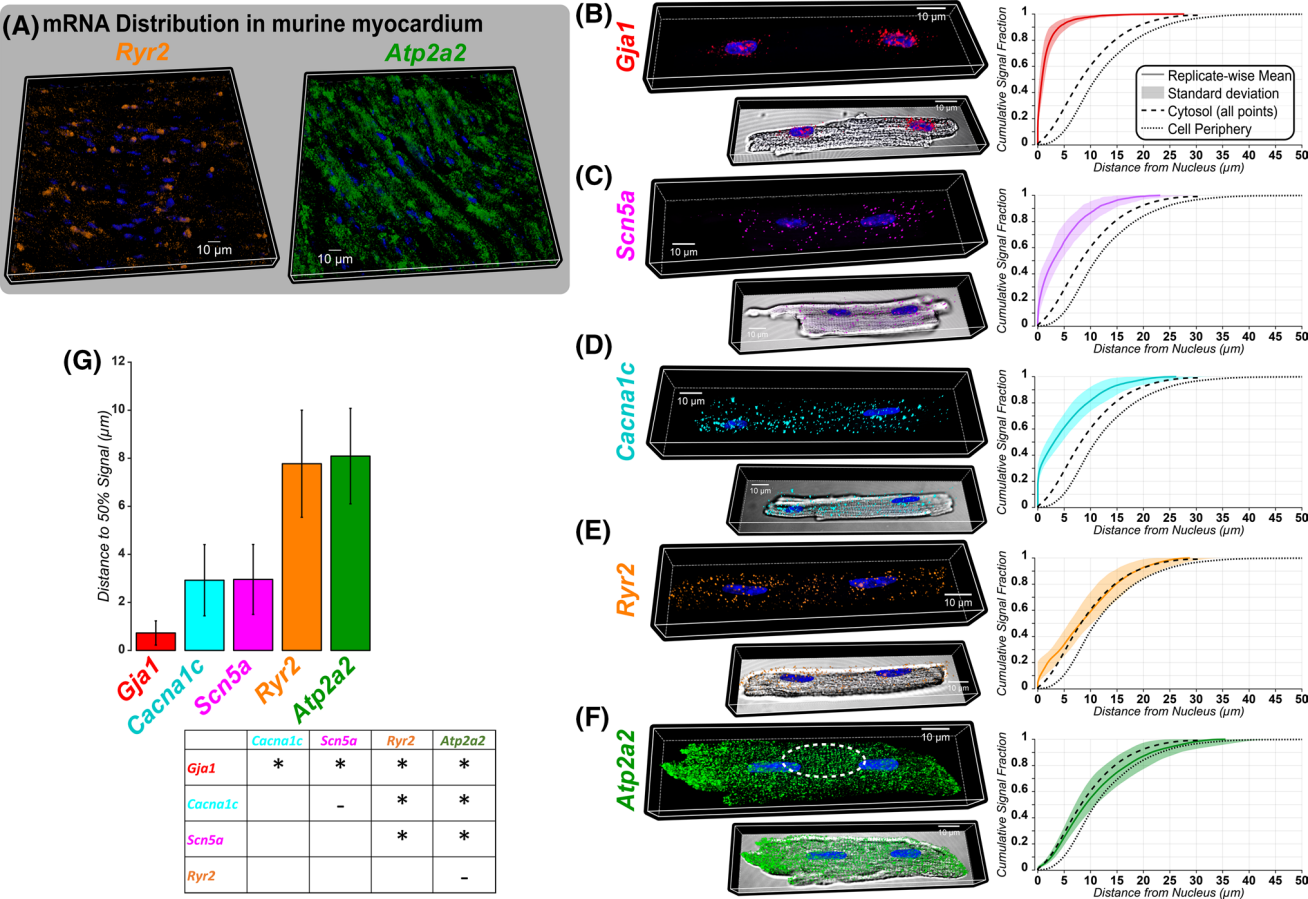
**A** Representative 3D confocal images of tissue sections from adult mouse heart showing *Ryr2* [encoding RyR2], and *Atp2a2* [encoding SERCA2a] mRNA visualized by RNAScope. **B–F, ** left: Representative 3D confocal images of adult cardiac myocytes showing *Gja1* [encoding Cx43], *Scn5a* [encoding Na_V_1.5], *Cacna1c* [encoding Ca_V_1.2], *Ryr2* [encoding RyR2], and *Atp2a2* [encoding SERCA2a] mRNA visualized by RNAscope. The dashed white ellipse in F highlights striated distribution of *Atp2a2* mRNA. In-depth analysis of striated mRNA localization is included in Supplementary Fig. 5. **B–F, ** bottom: RNAscope signals overlaid on DIC (grayscale). RNAScope signals for all species tested are shown with and without DIC overlay in Supplementary Fig. 1. **B–F, ** right: Cumulative distribution functions of mRNA signal vs. distance from the nucleus with standard deviations indicated by shaded regions. Dashed and dotted black lines, respectively, show CDFs for all voxels located in the cytosolic space and voxels located at the cell periphery. **G** Summary plot of distance from the nucleus within which 50% of mRNA are located. Table: Results from Bonferroni-corrected Wilcoxon’s test. * *p* < 0.01,—*p* = ns. (*Gja1*: *n* = 10 cells from 3 hearts; *Scn5a*: *n* = 16 cells from 3 hearts; *Cacna1c*: *n* = 16 cells from 3 hearts; *Ryr2*: *n* = 16 cells from 3 hearts; *Atp2a2*: *n* = 15 cells from 3 hearts)

Despite significant progress in studying the structure and function of cardiac myocyte membrane transport proteins, fundamental aspects of their biology, vis-à-vis synthesis, processing, and trafficking remain poorly understood. To gain new fundamental insights into the production of cardiac membrane proteins, we examined the distribution of mRNA templates for synthesis of several key SL membrane proteins, Cx43 (encoded by *Gja1*), Na_V_1.5 (encoded by *Scn5a*), and Ca_V_1.2 (encoded by *Cacna1c*), and SR membrane proteins, RyR2 (encoded by *Ryr2*), and SERCA2a (encoded by *Atp2a2*), in the adult mouse heart. Fluorescence in situ hybridization capable of single-molecule sensitivity (RNAScope™) in combination with confocal microscopy enabled visualization of mRNA molecules as punctate fluorescent signals [16]. In murine myocardial tissue sections, this method revealed the presence of signals for *Ryr2* [RyR2 protein] and *Atp2a2* [SERCA2a protein] mRNA throughout the cytosolic space (Fig. 1A), suggesting the intriguing possibility that mRNA templates for the synthesis of these membrane proteins are distributed throughout cardiac myocytes to support on-site protein synthesis within their functional niches. To test this hypothesis, we undertook more in-depth studies in cardiac myocytes isolated from adult mouse ventricles (Fig. 1 B-F, left), which afford sufficient experimental control. The specificity of the RNAScope signals was demonstrated by negative control experiments in rat cardiac myocytes (Supplementary Fig. 2), where even homologous mRNA species were not detected by the engineered murine mRNA probes.

The SL membrane proteins Cx43 (encoded by *Gja1*) and Na_V_1.5 (encoded by *Scn5a*) are known to be preferentially localized to the intercalated disks (IDs) with the latter also found in surface sarcolemma and transverse tubules [56]. Following synthesis in the perinuclear region, these proteins are thought to move to the IDs via the canonical secretory protein trafficking (SPT) pathway [3, 20, 25]. In accordance with this notion, mRNAs for these proteins, especially *Gja1*, appeared to localize mainly to the nuclei and perinuclear regions of myocytes (Fig. 1B, C, left). On the other hand, Ca_V_1.2 (encoded by *Cacna1c*) localizes to the transverse tubules, while SERCA2a (encoded by *Atp2a2*) and RyR2 (encoded by *Ryr2*) localize to SR membranes [4, 27]. Previous studies suggested that trafficking of these proteins may also rely on the canonical SPT mechanism [3, 25, 44, 62]. Surprisingly, mRNA for several of these proteins, in addition to being present in and around the nuclei, exhibited cell-wide distribution (Fig. 1D-F, left). This pattern was most pronounced for the *Atp2a2* [encoding SERCA2a] signal that was widely distributed throughout the myocyte. We applied a distance transform-based approach to quantitatively assess the spatial distribution of RNAScope signals relative to the nuclei. Cumulative distribution functions (CDFs) of the shortest distance from the nuclear perimeter provide convenient parameterization of mRNA localization within the myocyte (Fig. 1B-F, right). In each case, the CDFs for all voxels within the cytosolic space (Fig. 1B-F; dashed black lines) and for those located at the cell periphery (Fig. 1B-F; dotted black lines) are overlaid to provide context. Whereas the CDF for any mRNA species uniformly distributed throughout the cytosol would align with the all-points cytosolic CDF, the CDF for the cell periphery indicates the outer limit for any intracellular signals. The CDFs (Fig. 1 B-F, right) and distances to 50% signal abundance (Fig. 1G) suggest that mRNAs for cardiomyocyte SL and SR membrane ion transport proteins follow three spatially distinguishable distribution patterns: perinuclear (*Gja1* [Cx43]), cell-wide (*Atp2a2* [SERCA2a], *Ryr2* [RyR2]), and an intermediate pattern (*Cacna1c* [Ca_V_1.2], *Scn5a* [Na_V_1.5]).

The abundance of mRNA normalized to cytosolic volume for these species was lowest for *Scn5a* [Na_V_1.5] and highest for *Atp2a2* [SERCA2a], with the other species at intermediate levels (Supplementary Fig. 3). We examined mRNA distribution in the cytosolic space between nuclei, since this region is especially rich in ER and Golgi structures. Although the mRNA signal density in the internuclear space was higher compared to the rest of the cytosol (but lower than within the nuclei), the remaining cytosolic space accounted for much greater mRNA signal mass relative to the internuclear space (Supplementary Fig. 4A). Furthermore, inclusion or exclusion of the internuclear space did not appreciably alter the spatial distribution of cytosolic mRNA signals for any of the species tested (Supplementary Fig. 4B). Intriguingly, in most myocytes, *Atp2a2* mRNA [encoding SERCA2a] displayed a cross-striated pattern of organization (Fig. 1F, dashed white ellipse; Supplementary Fig. 5A-E). Comparison of intensity profiles for *Atp2a2* mRNA fluorescence and transmitted light signals revealed that *Atp2a2* mRNA were preferentially concentrated along light bands, consistent with network SR structures (Supplementary Fig. 5C). Indeed, *Atp2a2* mRNA displayed a periodicity of 1.65 µm (Supplementary Fig. 5D), consistent with characteristic sarcomere spacing [4, 63]. Of note, mRNA for myosin VI (*Myh6*), one of the principal components of the contractile apparatus in small rodents, had similar localization, albeit without significant overlap with *Atp2a2* (Supplementary Fig. 5A, B). Taken together, these results suggest that mRNAs for cardiomyocyte SL and SR membrane ion transport proteins may have distinct mechanisms for trafficking, synthesis, and processing: i) centralized, consistent with the SPT doctrine, and ii) novel, distributed—widely dispersed throughout the cell, yet integrated into myocyte functional units, sarcomeres. We termed the latter mechanism “distributed” to highlight its differences from centralized protein synthesis (as per canonical SPT) and potentially, also from local protein synthesis (occurring within morphologically distinct sub-cellular niches).

### Molecular machinery for SL/SR membrane protein synthesis is distributed throughout the cardiac myocyte

**Fig. 2:**
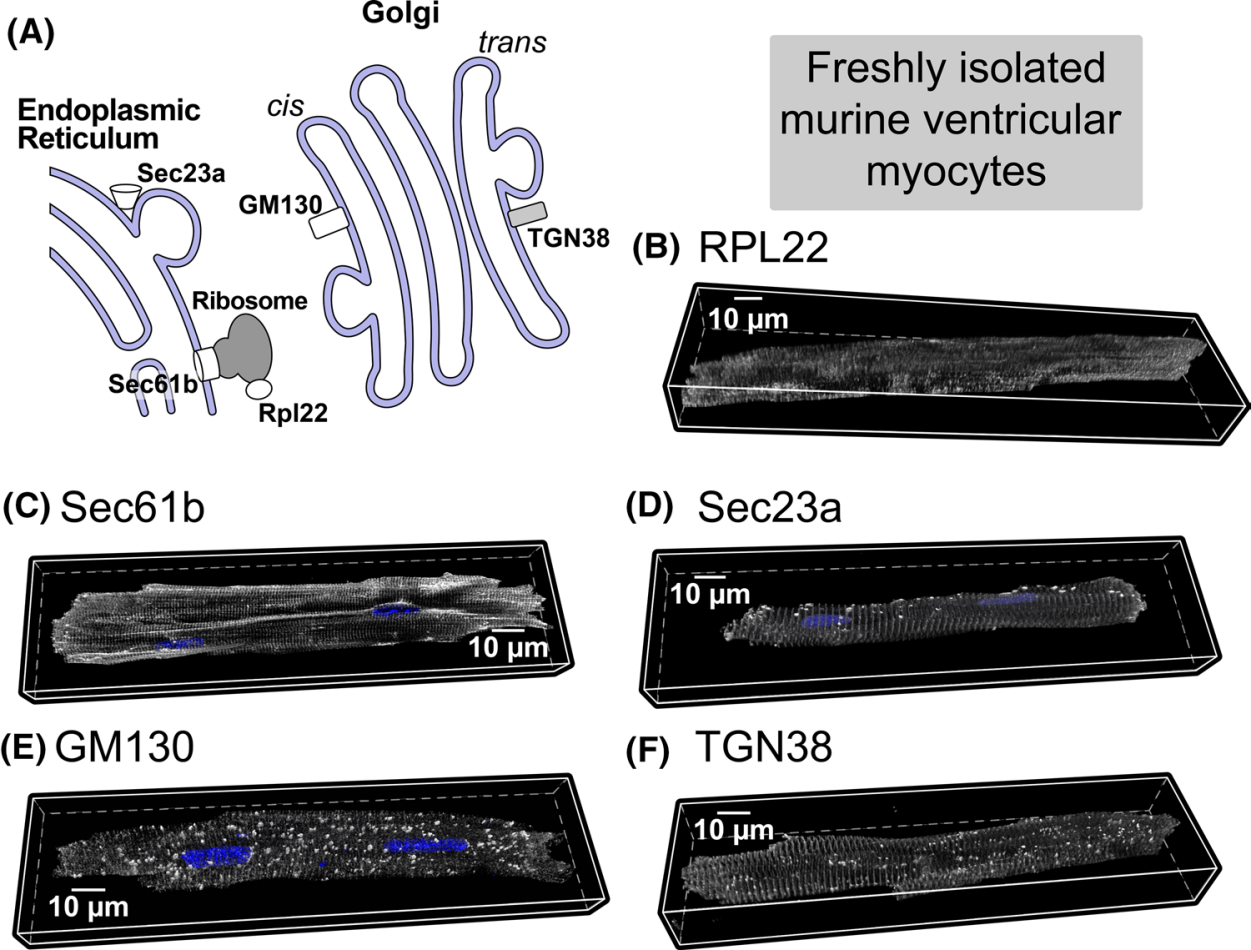
**A** Schematic of protein synthesis machinery. Representative 3D confocal images of adult cardiac myocytes showing **B** ribosomal protein Rpl22 (associated with actively translating ribosomes), **C** protein transport protein Sec61b, part of the translocon complex, **D** protein transport protein Sec23a, component of COPII, **E** trans-Golgi network protein TGN38, and **F** Cis-Golgi protein GM130. In all cases, proteins are shown in grayscale with nuclei in blue (3–9 cells per protein from 2 hearts). Quantitative analysis of these data and additional control experiments (along with n’s for specific experiments) are included in Supplementary Fig. 6. Antibodies used for these studies are listed in the methods

**Fig. 3:**
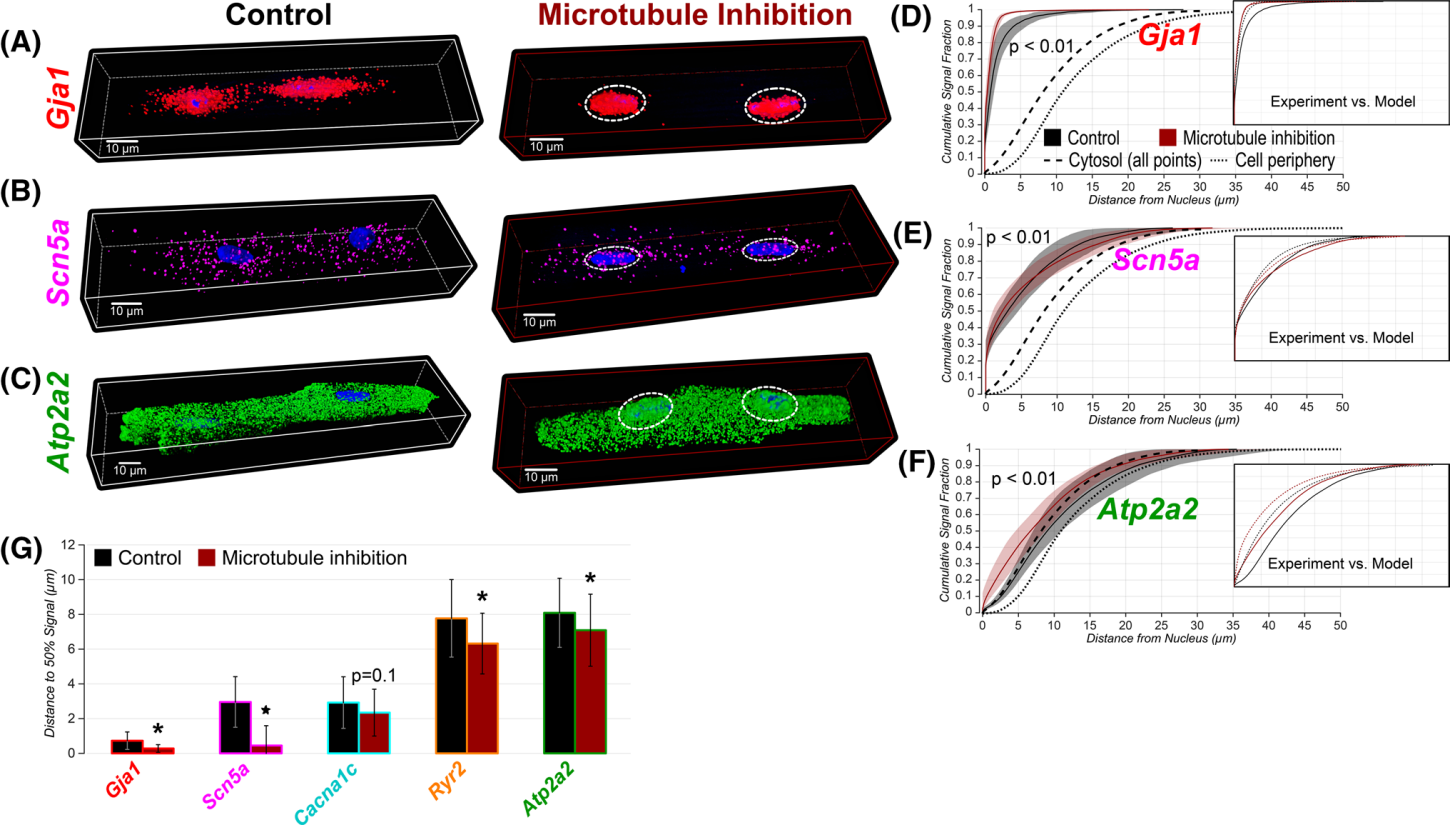
**A-C** Representative 3D confocal images of adult cardiac myocytes showing *Gja1*, *Scn5a*, and *Atp2a2* mRNA under control conditions (left) and following microtubule inhibition (right). White dashed ellipses around nuclei highlight perinuclear mRNA accumulation following microtubule inhibition. **D-F** Cumulative distribution functions of mRNA signal vs. distance from the nucleus under control conditions (white) and following microtubule inhibition (red). Dashed and dotted black lines, respectively, show CDFs for all voxels located in the cytosolic space and voxels located at the cell periphery. Inset panels: solid lines show experimental data, and dashed lines show model results. p values indicated are from two-sample Kolmogorov–Smirnov tests. **G** Summary plot of distance from the nucleus within which 50% of mRNA are located. * *p* < 0.01 (Bonferroni-corrected Wilcoxon’s test). (Control: *Gja1* [Cx43]: *n* = 10 cells from 3 hearts; *Scn5a* [Na_V_1.5]: *n* = 16 cells from 3 hearts; *Cacna1c* [Ca_V_1.2]: *n* = 16 cells from 3 hearts; *Ryr2* [RyR2]: *n* = 16 cells from 3 hearts; *Atp2a2* [SERCA2a]: *n* = 15 cells from 3 hearts; Microtubule inhibition: *Gja1*: *n* = 10 cells from 3 heart; *Scn5a*: *n* = 15 cells from 3 hearts; *Cacna1c*: *n* = 15 cells from 3 hearts; *Ryr2*: *n* = 15 cells from 3 hearts; *Atp2a2*: *n* = 16 cells from 3 hearts)

The classical SPT mechanism dictates that membrane proteins should be synthesized in the perinuclear regions. However, the observed cell-wide distribution of mRNA for SR/SL proteins suggests that they may be synthesized locally at sites of functional utilization within cardiac myocytes. Per the SPT mechanism, translation of membrane protein mRNA occurs on ribosomes of the rough ER with further processing in the Golgi complex, all occurring within the perinuclear space. However, the previous studies have identified ribosomes [48] and Golgi components [2] throughout the cytosolic space in cardiac myocytes. We examined the localization/distribution of these structures, via their key components (Fig. 2A), in freshly isolated murine cardiac myocytes using confocal microscopy. As expected, immunostaining for Rpl22, a marker of polysomes (actively translating ribosomes) [47], was found throughout the myocyte with cross-striated patterns consistent with sarcomeric structure (Fig. 2B). Inconsistent with SPT, immunosignals for the proteins Sec61b (part of the translocon complex; Fig. 2C) and Sec23a (COPII vesicles; Fig. 3D), which are requisite components of membrane protein translation and processing, were distributed cell-wide, also in a cross-striated pattern, while staining for the medial Golgi protein, GM130, displayed a cell-wide scattered, punctate distribution (Fig. 2E), in agreement with Golgi components’ localization previously observed for cardiac cells [2, 17] Notably, immunoreactivity for the trans-Golgi protein, TGN38, also displayed a cell-wide, cross-striated sarcomeric staining pattern (Fig. 2F). In all cases, CDFs of immunosignal distribution relative to the cell periphery closely followed the CDFs for all voxels within the cytosolic space, consistent with cell-wide distributions (Supplementary Fig. 6A, D). Furthermore, all proteins examined, except GM130, showed a peak on the Fourier transform power spectrum between 0.5 and 0.55 µm^−1^ (corresponding to 1.8–2 µm spacing), consistent with sarcomeric spacing. Similar patterns of localization (except for a small, albeit statistically significant, shift in GM130 localization) were observed for all proteins tested in myocytes cultured for 24 h (Supplementary Fig. 6B, D, E), indicating the stability of our cultured myocyte preparations. Similar results were obtained with a second set of antibodies against the same protein synthesis machinery (data not shown). Taken together, these results demonstrate that the requisite molecular machinery for synthesis and processing of membrane proteins is present throughout the cardiac myocyte cytosolic space. In particular, they highlight regions near sarcomeres as previously unappreciated hubs of membrane protein synthesis in cardiac myocytes.

### Cell-wide distribution of membrane protein mRNA utilizes microtubule trafficking

Local supply of mRNA for on-site protein synthesis in cardiomyocytes is expected to require a robust transport system for moving mRNA from the nuclei to sites of translation. To address this question, we sought to gain deeper understanding of the observed mRNA distributions. To this end, we quantified the change in mRNA signal concentration (% volume occupied by mRNA signal) as a function of distance from the nucleus. For SL protein mRNA, signal concentration was highest near the nucleus and declined while moving toward the cell periphery (Supplementary Fig. 7). In contrast, SR membrane protein mRNA showed increasing concentration while moving away from nuclei. These results, which are inconsistent with simple diffusion, pointed to a role for an active transport mechanism in distributing membrane protein mRNA.

Microtubules form a broad cytoskeletal network and are known to provide cargo transport functions throughout the cell, including trafficking of mRNA for cytosolic proteins [8, 18, 21]. To examine their role in trafficking membrane protein mRNA, we tested the effects of a microtubule inhibitor (colchicine; 10 µM; 8 and 24 h) on the cellular distribution of mRNA. Microtubule inhibition (8 h) resulted in redistribution of the signals for all mRNA species tested from the cytosol at large toward the perinuclear region (Fig. 3). This redistribution was most pronounced for *Scn5a* and *Gja1* mRNA [encoding Cx43], with more modest effects observed on *Cacna1c* [Ca_V_1.2], *Ryr2* [RyR2], and *Atp2a2* [SERCA2a] mRNA. Of note, microtubule inhibition did not significantly alter cell-wide (nuclear + cytosolic) abundance for any of the mRNA species; however, it increased mRNA abundance within the nucleus at the expense of abundance in the cytosol (Supplementary Fig. 8). More pronounced effects of a similar nature were observed following 24 h of microtubule inhibition (Supplementary Fig. 9); however, this treatment did not appreciably alter localization (save for small, albeit statistically significant, shifts in Rpl22, Sec23a localization; Supplementary Fig. 6C, D) or periodic distribution (Supplementary Fig. 6C, G) of protein synthesis machinery. By contrast, pharmacological disruption of the acto-myosin machinery (cytochalasin-D; 10 µM; 24 h) did not shift mRNA distribution toward the nuclei (Supplementary Fig. 9), suggesting that acto-myosin cargo transport does not play a major role in trafficking these mRNAs away from nuclei. That microtubule inhibition did not fully eliminate cytosolic mRNA populations could reflect the long (> 24 h) half-lives of membrane protein mRNA [48]. Consistent with this notion, we did not observe a significant decline in the abundance of membrane protein mRNA (except *Gja1* [Cx43]) following 24 h treatment of myocytes with a transcription inhibitor (Supplementary Fig. 10—DRB). Overall, these data suggest that cell-wide distribution of membrane protein mRNA is determined, at least in part, by microtubule trafficking.

### Mechanism of trafficking varies between different mRNA species

To further probe the role of microtubule trafficking, we compared the experimentally observed cytoplasmic distributions of different mRNA species with results from a one-dimensional diffusion–advection model (Supplementary Fig. 11A), in which microtubule trafficking is represented by advection [12, 14]. This simple model closely recapitulated the cytosolic distributions of mRNA for SL membrane proteins (*Gja1* [Cx43], *Scn5a* [Na_V_1.5], and *Cacna1c* [Ca_V_1.2]) under control conditions (Supplementary Fig. 11 B-D, G), as well as during microtubule inhibition (Fig. 3 D-E, inset panels; Supplementary Fig. 11 B-D, G). However, the cytosolic distributions of mRNA for SR membrane proteins (*Ryr2* [RyR2] and *Atp2a2* [SERCA2a]) were shifted further away from the nuclei than predicted by the model (Fig. 3F, inset panel; Supplementary Fig. 11 E–G). Furthermore, removing advection from the model shifted all mRNA distributions to perinuclear regions (Supplementary Fig. 11H). These simulation results lend further support to a crucial role for microtubule trafficking in distributing mRNA cell-wide. These results are consistent with our experimental observations that CDFs for *Ryr2* [RyR2] and *Atp2a2* [SERCA2a] mRNA were closely aligned or even right shifted relative to the all-points cytosolic CDF and that concentrations of these mRNA increased while moving away from nuclei. Taken together, these data suggest that trafficking regulation for SR membrane protein mRNA may be more complex, potentially entailing additional mechanisms of transport, anchoring, or degradation compared to mRNA for SL membrane proteins.

### *Visualization of ribosome-associated mRNA with mRNA–rRNA proximity-ligated *in situ* hybridization (MR-PLISH)*

**Fig. 4:**
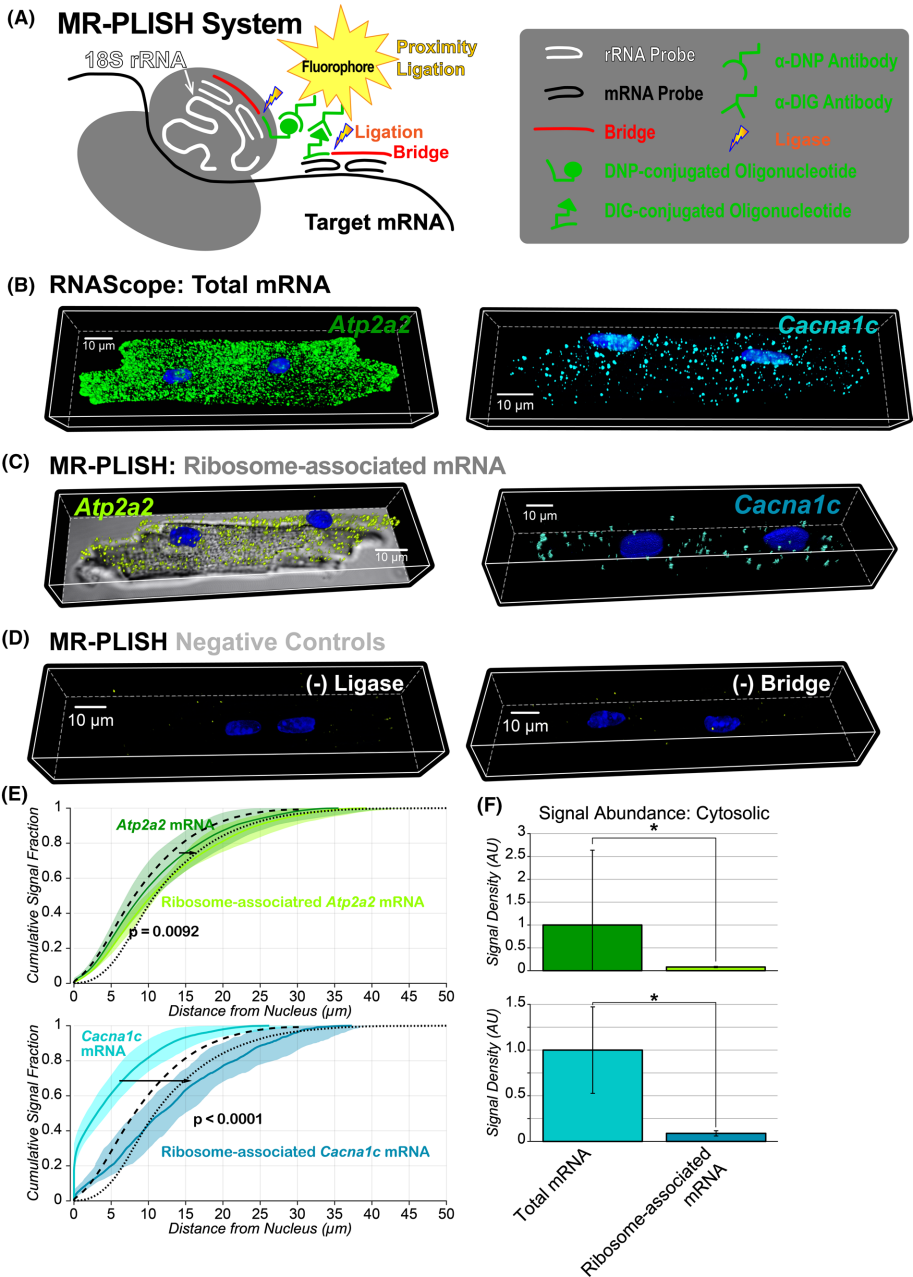
**A** Schematic of the MR-PLISH technique. Representative 3D confocal images of adult cardiac myocytes showing **B** total mRNA (RNAScope), **C** sites of active translation (MR-PLISH), and **D** results from negative control MR-PLISH experiments. **E** Cumulative distribution functions of total and ribosome-associated mRNA vs. distance from the nucleus. p values indicated are from two-sample Kolmogorov–Smirnov tests. Dashed and dotted black lines, respectively, show CDFs for all voxels located in the cytosolic space and voxels located at the cell periphery. **F** Summary plots showing abundance of total mRNA and active translation sites. * Wilcoxon’s test: *p* < 0.01. (RNAScope: *n* = 15 cells from 3 hearts; MR-PLISH: *n* = 12 cells from 3 hearts)

The aforementioned results suggest that several membrane proteins in cardiac myocytes may be synthesized on-site within functional niches. We sought direct evidence of such non-canonical synthesis of membrane proteins away from the nucleus. Therefore, we developed MR-PLISH (mRNA-rRNA Proximity Ligated In Situ Hybridization), a novel method for detection of a specific mRNA interacting with a translating ribosome. This method allows us to detect specific mRNAs with 18S ribosomal RNAs (*Rn18s*) if the distance between them is less then ~ 40 nm (Fig. 4A), selectively identifying ribosome-associated mRNA. As was the case with *Atp2a2* [SERCA2a] and *Cacna1c* [Ca_V_1.2] mRNA (Fig. 4B), ribosome-associated mRNA for these species (Fig. 4C) also displayed a cell-wide distribution. Negative control experiments performed in the absence of ligase or bridge, required for the MR-PLISH hybridization reaction, presented no appreciable signals (Fig. 4D). Additionally, pharmacologically induced mRNA–ribosome dissociation (puromycin, 200 µg/ml; 1.5 h) markedly decreased MR-PLISH signal abundance (especially in the cytosol), while preventing mRNA–ribosome dissociation using the translation–elongation inhibitor cycloheximide (50 µg/ml; 1.5 h) did not have an appreciable effect (Supplementary Fig. 12). As would be expected for distributed protein synthesis, both total and ribosome-associated mRNA for *Atp2a2* [SERCA2a] and *Cacna1c* [Ca_V_1.2] displayed similar cytosolic distributions, with ribosome-associated mRNA shifted further from the nucleus (Fig. 4E). However, cytosolic abundance of ribosome-associated mRNA for *Atp2a2* [SERCA2a] and *Cacna1c* [Ca_V_1.2] was, respectively, ~ 12.5- and 11.3-fold lower than the corresponding total mRNA (Fig. 4F), suggesting that ~ 8% of *Atp2a2* mRNA [encoding SERCA2a] and ~ 9% of *Cacna1c* mRNA [encoding Ca_V_1.2] are associated with ribosomes (thus, potentially being translated) at any given time. These results further support the notion that active synthesis of SERCA2a (encoded by *Atp2a2* mRNA) occurs throughout the cardiac myocyte.

**Fig. 5:**
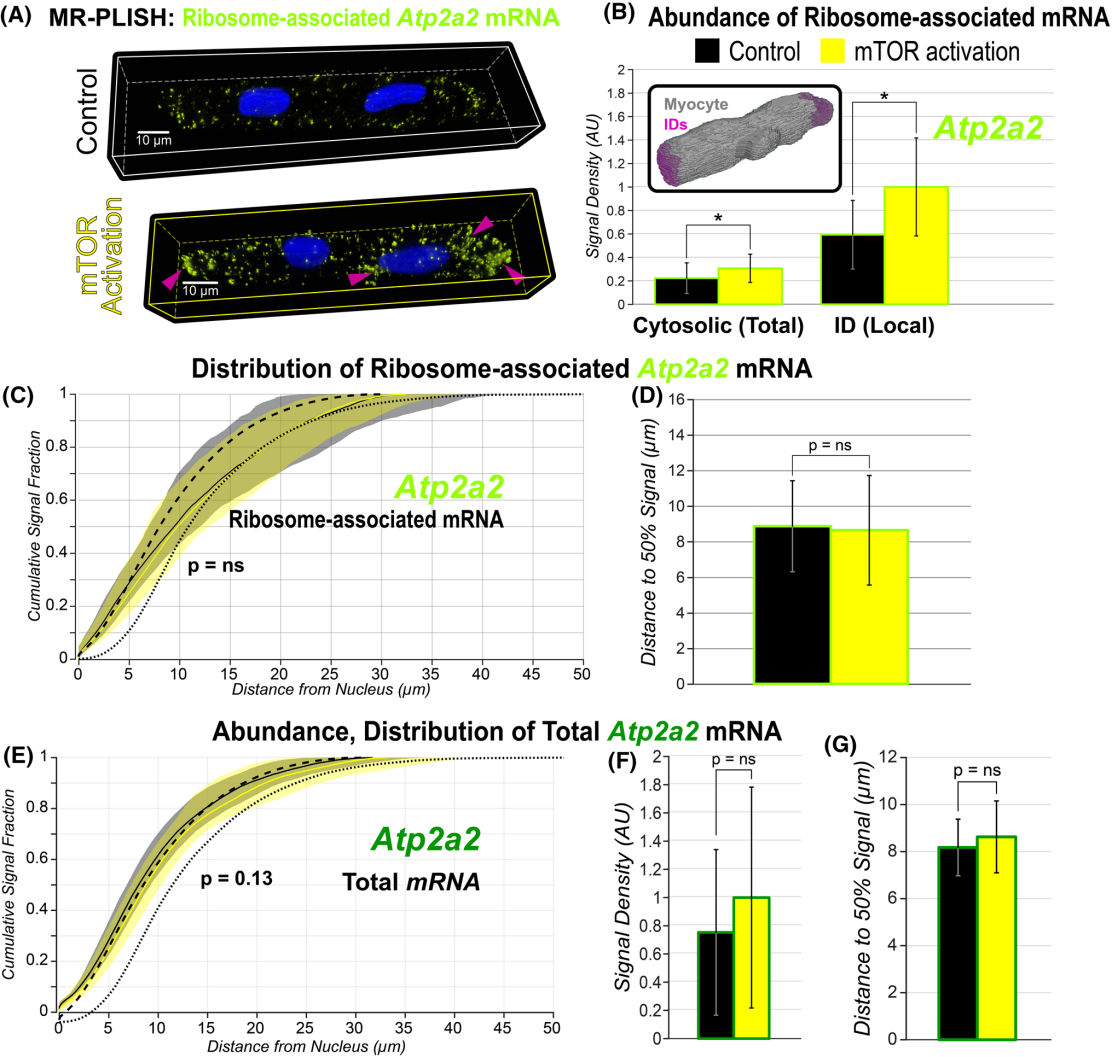
**A** Representative 3D confocal images of adult cardiac myocytes showing ribosome-associated *Atp2a2* mRNA under control conditions (top) and following mTOR activation (bottom). **B** Abundance of ribosome-associated *Atp2a2* mRNA (encoding SERCA2a) throughout the cytosol and at IDs. * Wilcoxon’s test: *p* < 0.01 Inset: Representative image showing segmentation of myocyte (gray) and IDs (pink). Inset panel illustrates segmentation of ID regions. **C** Cumulative distributions and **D** distance from nucleus to 50% signal for ribosome-associated *Atp2a2 mRNA* (MR-PLISH signals). **E** Cumulative distributions, **F** abundance, and **G** distance from nucleus to 50% signal for *Atp2a2* mRNA (RNAScope signals). Results during mTOR activation are shown in yellow with control data in black. Dashed and dotted black lines, respectively, show CDFs for all voxels located in the cytosolic space and voxels located at the cell periphery in C and E. *p* value indicated in panels C and E are from two-sample Kolmogorov–Smirnov tests while those in D, F, and G are from Wilcoxon tests. (*n* = 21 cells / group from 3 hearts)

The mTOR pathway is an established regulator of mRNA translation and proteins synthesis in various cell types, including cardiac myocytes [43], and has been shown to regulate local protein synthesis in neurons [43]. To further validate our MR-PLISH technique, we tested whether signals corresponding to ribosome-associated mRNA were upregulated following mTOR activation. Indeed, mTOR activation (MHY1485; 2 µM; 3 h) increased *Atp2a2* (SERCA2a) MR-PLISH signal abundance throughout the cytosol (Fig. 5A-D). Signal abundance was especially enhanced at cell end sites, consistent with intercalated disks (IDs; Fig. 5B, Supplementary Fig. 13). In contrast, pharmacological mTOR activation did not appreciably alter the abundance or cellular distribution of total *Atp2a2* mRNA (encoding SERCA2a; Fig. 5E-G). These data provide further validation of MR-PLISH as a means to selectively and specifically detect ribosome-associated mRNA.

### Distribution of sarcolemmal (SL) and sarcoplasmic reticulum (SR) membrane protein mRNA in human myocardium

To test whether the murine findings are evolutionarily conserved, we assessed the distribution of membrane protein mRNA in human myocardium. Consistent with our results from murine myocardium and isolated myocytes, we observed *RYR2* (RyR2), *ATP2A2* (SERCA2A), and *SCN5A* (Na_V_1.5) mRNA throughout the cytosolic space in both non-failing and failing human myocardium (Supplementary Fig. 14). These observations suggest that distributed synthesis of membrane proteins may play an important role in the human heart in both health and disease. Quantitative analysis revealed a significant increase in the density of *SCN5A* (Na_V_1.5) mRNA puncta in failing myocardium in comparison to non-failing controls (Supplementary Fig. 14D). These data extend our findings regarding distributed synthesis from the murine heart to human.

## Discussion

In this study, we examined how protein synthesis is spatially organized within cardiac myocytes for membrane proteins with key roles in electrical excitability and contractility localized to both the sarcolemma (SL; namely Ca_V_1.2, and Na_V_1.5) and sarcoplasmic reticulum (SR; namely SERCA2a and RyR2). It is commonly held that all membrane proteins in cardiac myocytes are synthesized and processed in ER/Golgi, in the perinuclear regions, and then transported to locations of deployment in the SL and SR membranes (i.e., the SPT pathway) [3, 20, 25]. In contrast to such centralized mechanisms, we present evidence for membrane protein synthesis occurring throughout the cardiac myocyte cytosolic space, providing on-site supply of membrane proteins for local use. We dub this new type of decentralized membrane protein synthesis “distributed” to highlight its differences not only from centralized synthesis (as per canonical SPT) but also potentially from ‘local’ synthesis as occurs within structurally distinct niches in cells like neurons [24, 26].

### Distributed synthesis of key cardiac membrane proteins

In support of the distributed protein synthesis mechanism, we observed that, in the mouse heart, mRNA and the requisite molecular machinery for synthesis of the aforementioned membrane proteins, including the translocon, SR-embedded ribosomes and *cis* and *trans-*Golgi, exist not only around the nuclei, but are distributed throughout the entire cytosolic space in a striated pattern consistent with sarcomeric organization. We further demonstrate that microtubule trafficking provides mRNA for distributed protein synthesis. We applied a novel in situ hybridization assay, which we term MR-PLISH, to directly visualize individual ribosome-associated mRNA (which identify potential sites of active translation). This technique indicated the presence of ribosome-associated *Atp2a2* (SERCA2a protein) and *Cacna1c* (Ca_V_1.2 protein) mRNA throughout the cardiac myocyte. Moreover, in the case of the most abundant species we studied, *Atp2a2* (SERCA2a)*,* mRNA were preferentially localized in a striated pattern, matching sarcomeric spacing, at sites consistent with network SR. Taken together, our results suggest that sarcomeric units within myocytes may include membrane protein synthesis machinery as integral components, making them modular units of cardiac myocyte biology as well as physiology. Finally, we found that distributed mRNA pools are present in both normal and pathologically hypertrophied (failing) human myocardium, extending our findings from the mouse to human. These results show, for the first time, that synthesis of SL and SR membrane proteins in cardiac myocytes occurs on-site from dedicated mRNA pools with potential roles in both health and disease.

### Three types of membrane protein mRNA distribution

It is commonly assumed that the perinuclear synthesis and trafficking of membrane proteins in cardiac myocytes follows the classical secretory pathway (rough ER/SR → Golgi → trafficking to membrane) determined from typical mammalian cells, while cytosolic proteins can be synthesized throughout the cell [3, 20, 25, 33]. A handful of studies examining trafficking of sarcolemmal proteins including Cx43 [49, 51], Ca_V_1.2 [3, 28, 29], and Na_V_1.5 [9, 36, 46] have indeed suggested that these proteins originate in the perinuclear region, from which they are transported to the surface membrane including intercalated disks (IDs). Consistent with this notion, we report perinuclear localization of *Gja1* mRNA [corresponding to Cx43 protein]. However, Cx43 may be the exception to the rule, given that we found other membrane protein mRNA distributed throughout the cytosolic space. Through both experimental disruption of microtubules as well as mathematical modeling, we demonstrate a role for active bio-transport in maintaining local mRNA pools. Overall, our results suggest that there are at least three schemes for the distribution of membrane protein mRNA in cardiac myocytes: (i) perinuclear retention of mRNA, consistent with centralized synthesis (Cx43); (ii) cell-wide distribution of mRNA, consistent with distributed protein synthesis (SERCA2a, RyR2), and (iii) a mixed arrangement combining features of both aforementioned schemes (Ca_V_1.2, Na_V_1.5). Notably, as detected by MR-PLISH, the distribution of ribosome-associated mRNAs for SERCA2a and Ca_V_1.2 were ~ tenfold lower in abundance than total mRNAs with distributions shifted further toward the myocyte periphery. These results suggest that a significant fraction of observed mRNA transcripts are stored near nuclei or captured *en route* to on-site translation. These results may suggest that centralized synthesis is reserved by the cell for ID membrane proteins such as Cx43, whereas distributed protein synthesis is utilized for the production of SR/T-tubule proteins such as RyR2 and SERCA2a. The mixed type of protein synthesis may apply to proteins localized to both ID and T-tubule membranes. Further experiments are needed to test these possibilities.

### Membrane protein mRNA trafficking involves microtubules

Whereas centralized protein synthesis requires cytoskeletal trafficking of synthesized proteins (as demonstrated for Cx43, Na_V_1.5, and K_ir_2.1 [15, 36, 42, 49, 50, 57]), distributed protein synthesis utilizes cytoskeletal transport to distribute mRNAs to sites of translation [33, 48]. We demonstrate that microtubule inhibition (but not acto-myosin disruption) shifted the distribution of membrane protein mRNA toward the nucleus. Thus, our work suggests that microtubule mRNA transport, previously demonstrated for cytosolic proteins [48], is also important for distributed synthesis of membrane proteins. Intriguingly, mRNA for SR proteins were found cell-wide with distributions shifted further away from nuclei than predicted by simple diffusion–advection. Indeed, our analyses revealed preferential enrichment of these mRNA at the cell periphery, indicative of more complex, multi-modal transport mechanisms. Recently, He and colleagues [25] showed that virally transfected SERCA is synthesized in the perinuclear region and then spreads cell-wide via microtubule trafficking of vesicles along the SR network. However, they only examined localization of SERCA protein (and not mRNA); thus, it is unclear whether their observations reflect trafficking of synthesized protein or of mRNA, with subsequent on-site translation. Equally, any apparent divergence between their results and ours could be attributed to differences in processing of virally transferred DNA vs. native nuclear DNA in the heart. For example, targeting and trafficking of native mRNA involve untranslated regions (UTRs) of mRNA [7, 41] absent in exogenously derived mRNA. Future work and further technological innovation will be necessary to address such questions through domain-swap studies, which are currently not feasible in adult cardiac myocytes.

### Distributed vs local membrane protein synthesis

**Fig. 6:**
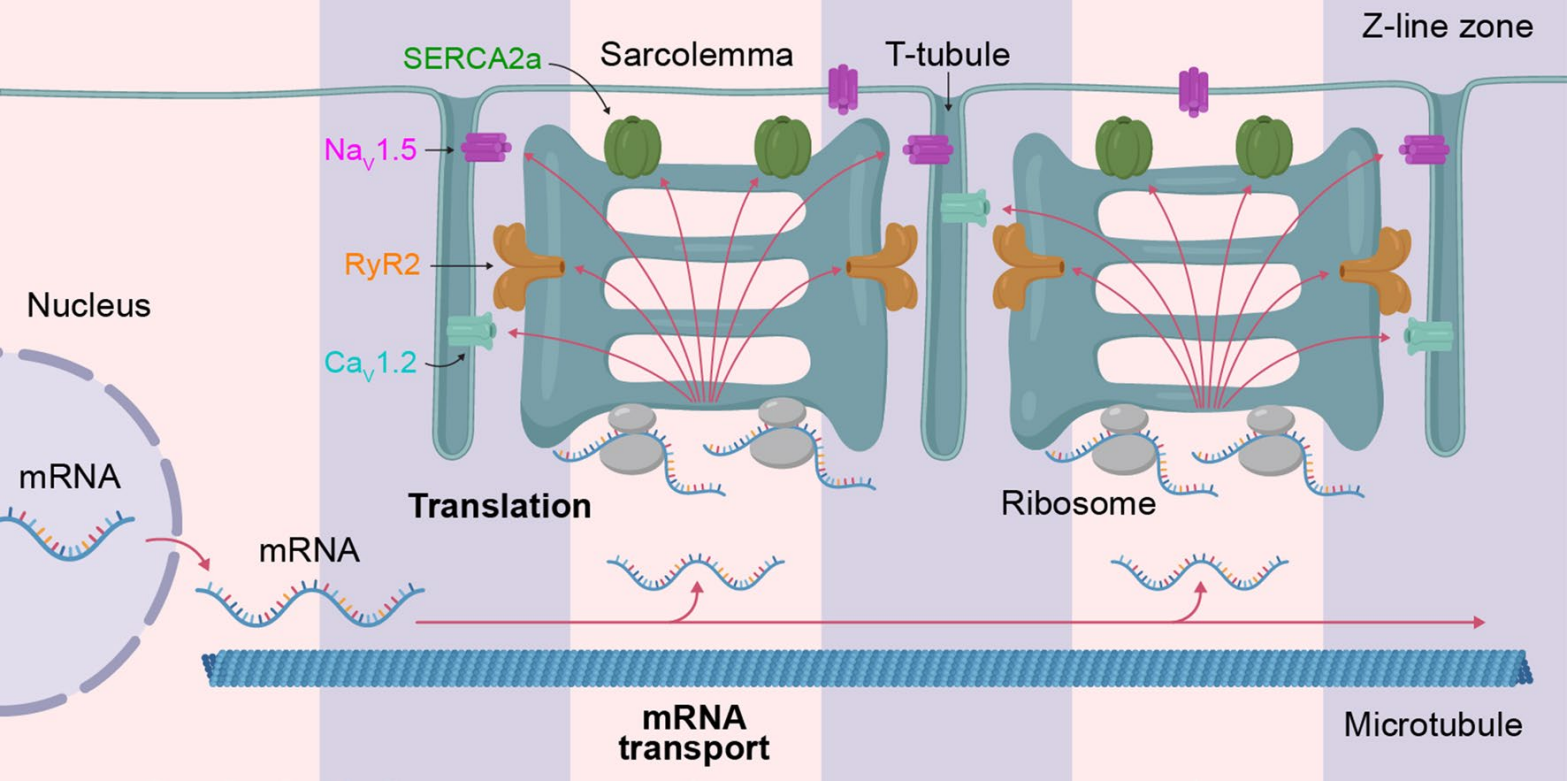
Schematic illustrating distributed protein syntehsis mechanisms in the cardiac myocyte

We demonstrate here for the first time that several membrane proteins in cardiac myocytes are synthesized in a decentralized manner (Fig. 6). However, decentralized synthesis of membrane proteins is not unique to cardiac myocytes. In neurons, key ion channel and receptor proteins are synthesized locally in axon end segments and dendrites with direct relevance to functions such as synaptic plasticity and learning [30, 31]. Moreover, analogous to our demonstration of mTOR-dependent increase in ribosome-bound (but not total) *Atp2a2* mRNA (encoding SERCA2a) abundance in cardiac myocytes, membrane protein synthesis in neuronal dendrites is regulated through the MAPK–mTOR pathway [43]. Despite these similarities, distributed protein synthesis in cardiac myocytes bears important differences from local synthesis in neurons. Whereas in neurons, on-site protein synthesis is truly *local* to specialized cellular outgrowths, such as dendrites or axon heads, in cardiac myocytes membrane protein synthesis is distributed throughout the entire myocyte volume, albeit localized to functional units (sarcomeres). Distributed synthesis in the large but modular cardiac myocytes and local synthesis in neurons with structurally heterogeneous domains may represent the same decentralized mechanism viewed through the distinct morphological prisms of these cell types. However, the possibility cannot be presently excluded that these are two disparate mechanisms involving different proportions of central vs. local control. Should further work point to the latter scenario, it will need to be determined whether neuron-like local protein synthesis occurs in more peripheral cardiac myocyte domains such as the ID and if cardiac-like distributed membrane protein synthesis occurs throughout the neuronal space. Future research into the mechanisms that regulate distributed synthesis will be necessary to answer these questions.

### Implications for normal biology and disease

Sarcomeric compartmentalization along with local signaling is recognized principles of structure–function organization in cardiac myocytes. Our data suggest that membrane protein synthesis may be an intrinsic part of this compartmentalized arrangement. In particular, we show that distributed pools of mRNA for SERCA2a (*Atp2a2)* are localized at sites consistent with network SR along with pools of mRNA for myosin VI (*Myh6).* In general, distributed protein synthesis confers some of the same advantages as localized synthesis does for neurons [24]. On-site control of protein synthesis obviates the need to transport each protein molecule to its site of deployment. Rather, a few mRNA molecules delivered to a site can be translated at locally determined rates to match functional protein expression to local demands. Moreover, proteins synthesized on-site can be put to immediate use, minimizing chances of degradation or undesirable post-translational modifications. The advantages of distributed membrane protein synthesis may be especially evident under conditions of physiological and pathological stresses when cardiac myocytes undergo hypertrophy. Hypertrophic myocyte growth involves genesis of new sarcomeres through new protein synthesis, resulting in up to 3–fourfold increase in cellular volume in human end-stage HF [19, 40]. The biological efficiency of distributed protein synthesis is expected to ease energetic demands associated with centralized synthesis and distribution of membrane proteins throughout the growing/enlarged myocyte. Consistent with this notion, we found mRNAs for key SR (RyR2, SERCA2a) and SL (Na_V_1.5, Ca_V_1.2) membrane proteins distributed throughout the myocyte space in both non-failing and failing (drastically hypertrophied) human myocardium.

### Limitations

Using cutting-edge methods, we visualized mRNA for proteins such as SERCA2a with sarcomeric resolution. However, spatial resolution of in situ hybridization-based methods (RNAScope, MR-PLISH) is limited by signal amplification, which adds multiple fluorophore molecules at each detected molecular site. This limits the ability to localize these signals to precise nano-scale neighborhoods. MR-PLISH detects ribosome-associated mRNA, which may not directly correlate with active translation sites under certain conditions. However, currently, no methods are available to directly verify active translation occurring at sites where ribosome-associated mRNA are detected for a specific protein species. Additionally, we provide multiple control experiments demonstrating the specificity and selectivity of MR-PLISH. The ability of Fourier transform analysis to detect periodic distribution of RNAScope/MR-PLISH signals is proportional to their abundance. Thus, the apparent lack of periodic distribution for signals with low abundance may be due to insufficient spatial sampling of patterns present. Finally, our data implicate microtubules in trafficking of membrane protein mRNA. However, the principles of selective targeting mRNA are poorly understood with available data, indicating that they are more complex/elaborate than simple encryption in the liner sequence of mRNA UTR regions [26]. Precise delineation of these mechanisms is precluded by limitations of current technology. For instance, domain-swap studies or expression of hybrid mRNA requires large-scale manipulations with genomic DNA in adult myocytes, which are beyond current technical capabilities.

### Summary

In conclusion, we provide the first evidence that key SL and SR membrane proteins in cardiac myocytes are synthesized on-site at locations of functional deployment from distributed mRNA pools maintained by microtubule trafficking. Compared to the classical centralized view of protein synthesis and its regulation, the demonstrated decentralized mechanism provides previously unanticipated flexibility for regulation of membrane protein expression and biological self-organization in cardiac myocytes during development, health, and disease. In particular, this concept is relevant to understanding adaptations such as hypertrophy, which are predicated on new protein synthesis. Furthermore, distributed synthesis of membrane proteins hints at an unexpected level of diversity in arrangements for membrane protein synthesis between different cell types and possibly within specific cell types such as cardiac myocytes and neurons [24, 26], and motivates further research in other specialized cell types. Future studies are also needed to define the specific components of feedback loops that regulate distributed protein synthesis in the heart, the biological and physiological cues that drive them, and the implications for health and disease.

## Supplementary Information

Below is the link to the electronic supplementary material.

Supplementary file1 (PDF 6345 kb)

## Data Availability

Raw experimental data are backed up on OSU servers and will be shared freely on request.

## Code availability

Image analysis and simulation code will be shared via open forums such as Matlab Central File Exchange.
