# Supplementary material for: Distributed synthesis of sarcolemmal and sarcoplasmic reticulum membrane proteins in cardiac myocytes
Source: Basic Res Cardiol. 2021 Oct 28;116(1):63. doi: 10.1007/s00395-021-00895-3 (PMC8553722; doi:10.1007/s00395-021-00895-3)
Supplement: Supplementary file 1 — Supplementary file1 (PDF 6345 kb) [file 395_2021_895_MOESM1_ESM.pdf]

Supplementary Information for

Distributed synthesis of sarcolemmal and sarcoplasmic reticulum membrane proteins in cardiac myocytes

Vladimir Bogdanov*[1,2], Andrew M. Soltisz*[1,3], Nicolae Moise[1,2], Galina Sakuta[1,2], Benjamin Hernandez Orengo[1,2], Paul M.L. Janssen[1,2], Seth H. Weinberg[1,3], Jonathan P. Davis*[1,2], Rengasayee Veeraraghavan*[1,2,3], Sandor Györke*[1,2]

Corresponding authors:

Sandor Györke (Sandor.Gyorke@osumc.edu), Rengasayee Veeraraghavan (veeraraghavan.12@osu.edu), Jonathan P. Davis (davis812@osu.edu)

This PDF file includes:

Supplementary text

Figures S1 to S14

SI References

SUPPLEMENTARY METHODS

All animal procedures were approved by Institutional Animal Care and Use Committee at The Ohio State University and performed in accordance with the Guide for the Care and Use of Laboratory Animals published by the U.S. National Institutes of Health (NIH Publication No. 85-23, revised 2011).

Adult Mouse Cardiac Myocyte Isolation and Primary Culture: Cardiac myocytes were enzymatically isolated from 8-12 week-old male C57BL6 mice and maintained in primary culture using the methods described by Ackers-Johnson et al[1].

Pharmacological Perturbations: Myocytes were treated with the following pharmacological agents: microtubule inhibitor (colchicine, 10 µM, 8 hours), mTOR activator (MHY1485, 2 µM, 3 hours).

Fluorescent Immunolabeling: Cells were fixed in 2% PFA at room temperature (RT) for 8 minutes, washed 3 times in PBS for 5 minutes, and incubated in permeabilizing buffer (0.2% Triton X100, 1xTBS, 10% FBS) for 10 minutes. Then samples were treated with blocking buffer (5% BSA, 1xTBS, 0.1% tween 20) for 1h at RT. Samples were then incubated overnight at 4°C in primary antibodies diluted in blocking buffer (1:100). Following this, samples were washed 4 x 5 minutes in TBST (1x TBS + 0.1% tween 20) at RT. Subsequently, samples were labeled with donkey anti-rabbit AlexaFluor 647, donkey anti-mouse AlexaFluor 568 and donkey anti-goat AlexaFluor 488 secondary antibodies (Abcam) at 1: 1000 dilution in blocking buffer (2 hours at RT), then washed 4 x 5 minutes in TBST at RT and mounted in Prolong Gold antifade reagent with DAPI (Invitrogen, Rockford, IL).

Preparation for *in situ* hybridization: Cells cultured on 8mm coverslips were fixed in 4% PFA in 1x PBS + 0.1% Diethyl Pyrocarbonate (DEPC) for 30 min at RT, then rinsed with 1x PBS + 0.1% DEPC, followed by serial dehydration in 50%, 70% and 100% ethanol for 5 min each. Finally, samples were transferred to fresh 100% ethanol and stored at -20C for up to two weeks. Cells, thus stored, were sequentially rehydrated in 70% and 50% ethanol for 2 minutes, followed by 10 minute exposure to PBST (1xPBS in 0.1% DEPC with 0.1% Tween20). Hybridization and incubation steps were performed in a humidified, covered chamber. For each incubation step, we used 50-70µl solution per 8mm coverslip.

RNAscope: Individual mRNA molecules were visualized using RNAscope Multiplex Fluorescent Reagent Kit v2 (Advanced Cell Diagnostics Inc., Newark, CA), implemented per manufacturer-recommended protocols. Briefly, probes were hybridized to the mRNA species of interest, following which cells were incubated in a series of amplification reagents, before finally being labeled with fluorophores. Of note, we made the following minor changes to the manufacturer-recommended protocol. We used 0.1% DEPC water in place of nuclease-free water. Protease III was diluted 1:15 and TSA fluorophores were diluted 1:1500. RNAScope probes used standard manufacturer-provided sequences:

Gja1: Cat No. 486191; Target Region: 971 - 2026; NM_010288.3
Scn5a: Cat No. 429881; Target Region: 393 - 1729; NM_021544.4
Cacna1c: Cat No. 445451-C3; Target Region: 4846 - 6605; NM_009781.4
Ryr2: Cat No. 479981; Target Region: 735 - 1636; NM_023868.2
Atp2a2: Cat No. 454731; Target Region: 535 - 2533; NM_001110140.3
Myh6: Cat No. 506251-C3; Target Region: 48 - 5162; NM_001164171.1
Human *RYR2*: Cat No. 415831-C2; Target Region: 8981 - 9977; NM_001035.2
Human *SCN5A*: Cat No. 430281-C2; Target Region: 1063 - 1995; NM_198056.2
Human *ATP2A2*: Cat No. 555371; Target Region: 1160 - 2357; NM_170665.3

Messenger RNA – Ribosomal RNA Proximity-Ligated *In Situ* Hybridization (MR-PLISH): In order to visualize sites of active translation for a specific mRNA species, we developed MR-PLISH. Briefly, complementary nucleotide (nt) double probes were hybridized to the mRNA species of interest and 18S ribosomal RNA (Rn18s). These were connected via a barcode nucleotide bridge, which was then ligated to barcoded short 11nt DNA oligos: digoxigenin (DIG) –conjugated for mRNA and 2,4-Dinitrophenol (DNP) -conjugated for rRNA. Finally, these were labeled with anti-DIG and anti-DNP antibodies and a proximity ligation approach used to generate fluorophores *in situ* through a rolling circle polymerization reaction at sites where anti-DIG and anti-DNP antibodies occur within ~40 nm of each other.

Probe design: The probes were designed as described in Nagendran et al., 2018[4] with some changes. Each detection site was targeted with a pair of hybridization probes. The H and R probes were designed using NCBI BLAST to eliminate cross detection of mRNA that share more than 10nt of the probe sequences. For *Rn18s* (18s

ribosomal RNA), we produced 27 double probes in total, and for *Atp2a2* (encoding Serca2a; NM_009722.3) we produced 43 double probes in total. The mRNA probes were targeted to protein coding sequences. These probe sequences are provided in the enclosed spreadsheet.

Barcoding procedure: Barcoding as performed as described previously[4] with some changes. Instead of a circular nucleotide bridge, we used short 11nt DNA oligos conjugated to either digoxigenin (DIG) or 2,4-Dinitrophenol (DNP). DIG-conjugated oligos were complimentary to a 3'-flanker of mRNAs probe, DNP-conjugated oligos were complimentary to a 3'-flanker of Rn18s (NR_003278.3) probe. To the hybridization-probe buffer (HP buffer: 1M sodium trichloroacetate, 50 mM Tris pH 7.4, 5 mM EDTA, 0.2 mg/mL Heparin) were added 50nM of each probe 2.7μM *Rn18s* probes and 4.3μM *Atp2a2* probes in total. A bridge-barcode buffer (BB: 2% BSA, 0.2 mg/mL heparin, 0.05% Tween-20, 1x T4 ligase buffer in RNAse-free water) was prepared by mixing a bridge and DNP-, DIG-conjugated oligos in BB buffer at a final concentration of 6μM. Samples were incubated in HP buffer for 2h at 37C, washed 4 x 5 minutes at RT in HP buffer and incubated in BB buffer for 1h at 37C. They were then washed in PBST for 5 minutes at RT and incubated in ligation buffer for 1h at 37C. Then samples were washed 5 x 5 minutes in 2x SSC, 20% formamide, 0.1%, triton X100, 0.1% DEPC, 5 x 5 minutes in 2xSSC, 0.1%, Triton X100, 0.1% DEPC, and finally, 2 x 5 minutes in PBST.

Proximity Ligation: Anti-DIG and anti-DNP antibodies were diluted with 2% BSA in PBST at concentration of 1:100. Antibodies mix was added to cells and incubated overnight at 4C. Cells were washed 3 times per 5 minutes in PBST, then DuoLink kit was applied. Briefly: probes Plus and Minus were mixed with 2% BSA in PBST and incubated for 1h at 37C. Samples were washed 3 times per 5 minutes in Buffer A, ligase in ligation buffer was added, and cells were incubated for 30 minutes at 37C. After that cells were washed 2 times per 5 minutes in Buffer A, polymerase in Green buffer was added to cells and incubated for 3h at 37C. Then samples were washed 2 times for 5 minutes and mounted with DuoLink mounting media with DAPI.

Confocal Microscopy: Confocal microscopy was performed as previously described[3, 6]. Samples were imaged using an A1R-HD laser scanning confocal microscope equipped with four solid-state lasers (405 nm, 488 nm, 560 nm, 640 nm, 30 mW each), a 60x/1.4 numerical aperture oil immersion objective, two GaAsP detectors, and two high sensitivity photomultiplier tube detectors (Nikon, Melville, NY). Where multicolor imaging was performed, individual fluorophores were imaged sequentially with the excitation wavelength switching at the end of each frame. Additionally, in a subset of cases, a differential interference contrast image was collected concurrently via a transmitted light detector.

Image Analysis: Images were analyzed using morphological object localization (MOL), a custom algorithm implemented in Matlab (Mathworks Inc, Natick, MA). Briefly, the cell body, nuclei, and signal puncta were identified using object-based segmentation and an exact Euclidean distance transform was applied to calculate distances from mRNA signals to the outer perimeter of the closest nucleus. Distances thus measured were plotted as cumulative distribution functions (CDFs). Additionally, signal abundance was assessed as the ratio of the integral of normalized voxel intensities to the volume of the relevant compartment (nucleus, cytosol, or whole cell).

Image Segmentation: The cell body mask was generated by threshold all channels with high sensitivity then combining the resulting binary images using a pointwise logical AND operation and finally selecting the largest connected component as the cell body. The nuclear mask was generated by thresholding the nuclear channel with low sensitivity and selecting all connected components with a volume greater than 0.1% the volume of the cell body as nuclei. A morphological closing with a 30-voxel radius disk was applied to the body and nuclear mask for edge smoothing. The mRNA masks were generated by thresholding their respective channels with high sensitivity and selecting connected components with volumes greater than 20 voxels to help exclude noise. Cell end sites (consistent with intercalated disks) were segmented using morphological filtering with a line structure element aligned perpendicular to the long axis of myocytes.

Fluorescent Signal Localization: Fluorescent signals (RNAScope, MR-PLISH) were localized relative to cell nuclei using the distance transformation (DT) of the nuclear mask. The 3D exact Euclidean DT of the nuclear mask, an image where the value of each voxel is equal to that voxel's exact Euclidean distance from the nearest nuclear-positive voxel, was generated using a linear-time algorithm.[1] For a given cell, the set of mRNA-voxel distances was derived by extracting the subset of the nuclear DT which intersects the mRNA mask, thus outputting a distance measurement for each mRNA-positive voxel. Signal fraction as a function of distance from the nuclei was then plotted to produce the probability density function of an mRNA signal for an individual cell and cumulatively summed to produce the cumulative distribution function (CDF) for ease of interpretation and

statistical analysis. To combine the CDF data from multiple cells, the cumulative signal fraction (CSF) was quantized from 0 to 1 in increments of 0.001 and distances measurements were linearly interpolated to create common CSF data points between all cells which were mean-averaged to create a single plot. In these plots, lines represent the mean CSF for a group of cells as a function of distance from the nuclei and the shaded region around a line represents the CSF's standard deviation at each nuclear distance.

In order to further probe the distribution of fluorescent signals within the cytosol, we examined normalized signal concentration (% voxels occupied) as a function of normalized distance from the nucleus. Briefly, CDFs (Supplementary Fig. 4A) generated as described above were numerically differentiated to yield probability density functions (PDFs; Supplementary Fig. 4B) and the value of the PDF for each normalized distance value (0 being the edge of the nuclei and 1 being the cell periphery) was divided by the number of voxels available at that distance to yield normalized signal concentration (Supplementary Fig. 4C). In one approach, we performed linear regression fitting of signal concentration vs. distance from nuclei between normalized distances of 0.2 and 0.8 (Supplementary Fig. 4C – dashed lines) and evaluated the slope (Supplementary Fig. 4E). In a second approach, we numerically differentiated signal concentration as a function of normalized distance from the nuclei (Supplementary Fig. 4D) and evaluated its mean value between normalized distance of 0.2 and 0.8 (Supplementary Fig. 4F).

Other Plots: Fluorescent signal density is measured as the sum of all signal-positive voxel intensities in a given cellular compartment divided by the volume of that cellular compartment. Density measurements are normalized to the greatest density for a given plot. The signal histogram presents the voxel intensities (x-axis) of the segmented mRNA voxels normalized to the maximum voxel intensity of all gathered images. The signal fraction (y-axis) is presented as the mean ± the standard deviation.

Statistics: Pair-wise differences between localization CDFs was evaluated using the two-sample Kolmogorov-Smirnov test. Signal density is presented as the mean ± the standard deviation, and pair-wise differences were evaluated using the two-sample Wilcoxon rank sum test. An α value of 0.05 was used for all statistical tests. To test whether measured values different significantly from zero, the sign test was used with an α value of 0.05.

Mathematical Modeling: We modeled the transport of mRNA in the cytoplasm by the following 1D advection-diffusion partial differential equation:

$$\frac{\partial u_i}{\partial t} - D\frac{\partial^2 u_i}{\partial x} + a_i\frac{\partial u_i}{\partial x} = -d \cdot u_i,$$

where u_i is the concentration of mRNA *i*, *D* is the diffusion coefficient, a_i is a uniform velocity and d is the degradation rate. The equation is defined on the non-dimensional domain $[0,1]$, for which the boundary $x = 0$ represents the edge of the nucleus and $x = 1$ represents the cell membrane. Accordingly, we have the following boundary conditions:

$\frac{\partial u}{\partial x} = F_i$, for $x = 0$ and

$\frac{\partial u}{\partial x} = 0$, for $x = 1$,

where F_i is the mRNA in-flux into the domain.

Parameters were fit to experimental control data in two steps: 1. We first fit the mRNA in-flux F_i to the total amount of mRNA in the domain. We next fit a_i, the scalar uniform velocity, based on the experimental data for the signal based on distance from the nucleus.

For the colchicine case, we started by fitting a new inward flux, as total mRNA signal amount differs slightly between experiments. We simulate the spatial steady state (reached at time t_s) based on the prior fit velocity a_i. Then, for $t > t_s$, we set $a_i = 0$ (simulating the effects of colchicine on microtubule-assisted transport) and simulate an additional 8 hours.

The diffusion coefficient *D* is equal for all mRNA species. It was obtained by fitting the equation with $a_i = 0$ on the data for *Gja1*, which is least affected by the advection term (i.e. the fit is perfect with $a_{Gja1} = 0$). We assumed that the half-life of mRNA in the cytoplasm is 1 day for all species, hence $d = \frac{\ln 2}{1\ day} = 0.693/day$.

The partial differential equation was discretized using the backward Euler implicit scheme, with the diffusion term discretized with a centered stencil and the advection term discretized with the upwind stencil. All fitting steps were performed using nonlinear least squares curve fitting.

Reagents: For immunolabeling experiments, the following primary antibodies and probes were used:

1. DNA oligos (IDT, Coralville, IA); used for MR-PLISH
2. DIG conjugated DNA oligos (Eurofins Genomics, Louisville, KY); used for MR-PLISH
3. DNP conjugated DNA oligos (Eurofins Genomics, Louisville, KY); used for MR-PLISH
4. Digoxigenin, rabbit monoclonal, clone 9H27L19 (ThermoFisher Scientific, 700772); used for MR-PLISH at 1:100 dilution
5. Duolink™ *In Situ* PLA® Probe Anti-Goat PLUS (Sigma, DUO92003); used for MR-PLISH
6. Duolink™ *In Situ* PLA® Probe Anti-Rabbit MINUS (Sigma, DUO92005); used for MR-PLISH
7. Duolink™ *In Situ* Detection Reagents Green (Sigma, DUO92014); used for MR-PLISH
8. DNP, goat polyclonal (Bethyl, A150-117A); used for MR-PLISH at 1:100 dilution
9. Hi-T4™ DNA Ligase (New England Biolabs, M2622L); used for MR-PLISH
10. GM130, monoclonal, Clone 35/GM130 (BD, 610823)[5]; used for IF at 1:100 dilution
11. TGN38, sheep polyclonal (Bio-Rad Laboratories, AHP499G)[7]; used for IF at 1:100 dilution
12. RPL22, goat polyclonal (Novus Biologicals, NBP1-06069)[2]; used for IF at 1:100 dilution
13. Sec61b, rabbit polyclonal (Abcam, ab15576)[9]; used for IF at 1:100 dilution
14. Sec23a, rabbit polyclonal (Novus Biologicals, NBP2-34842)[8]; used for IF at 1:100 dilution
15. GM130, monoclonal conjugated Alexa Fluor® 647, Clone 35/GM130 (BD, 558712); used for IF at 1:100 dilution (additional confirmatory studies; data not shown)
16. COPII(Sec23a), rabbit polyclonal (ThermoFisher Scientific, PA1-069A); used for IF at 1:100 dilution (additional confirmatory studies; data not shown)
17. TGN46(TGN38), sheep polyclonal (ThermoFisher Scientific, PA1-84496); used for IF at 1:100 dilution (additional confirmatory studies; data not shown)
18. Sec61a, rabbit polyclonal (ThermoFisher Scientific, PA1-21773); used for IF at 1:100 dilution (additional confirmatory studies; data not shown)
19. Donkey anti-goat IgG H&L AlexaFluor® 488 (abcam, ab150133); used for IF at 1:1000 dilution
20. Donkey anti-rabbit IgG H&L AlexaFluor® 647 (abcam, ab150063); used for IF at 1:1000 dilution
21. Donkey anti-mouse IgG H&L AlexaFluor® 568 (abcam, ab175700); used for IF at 1:1000 dilution

SUPPLEMENTARY RESULTS

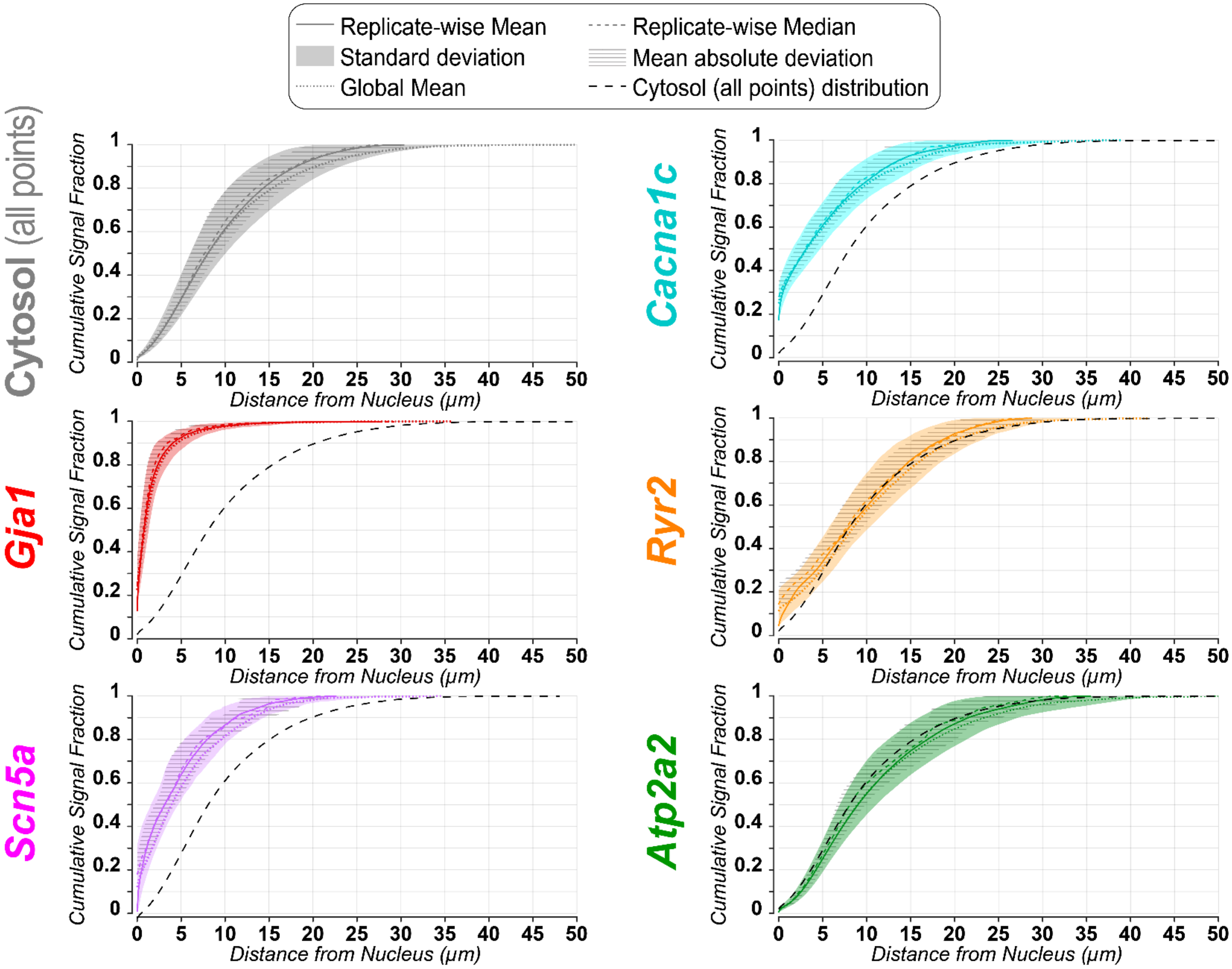

Figure S1. Cumulative distribution functions of mRNA signal vs. distance from the nucleus with standard deviations indicated by shaded regions. Comparison of mean CDFs ± SD with median CDFs ± median absolute deviation (MAD) and global CDFs (calculated by pooling distance measurements from all cells within each group). Dashed black lines show CDFs for all voxels located in the cytosolic space. (*Gja1* [Cx43]: n = 10 cells from 3 hearts; *Scn5a* [Na$_{V}$1.5]: n = 16 cells from 3 hearts; *Cacna1c* [Ca$_{V}$1.2]: n = 16 cells from 3 hearts; *Ryr2* [RyR2]: n = 16 cells from 3 hearts; *Atp2a2* [Serca2a]: n = 15 cells from 3 hearts)

A) RNAScope Negative Controls

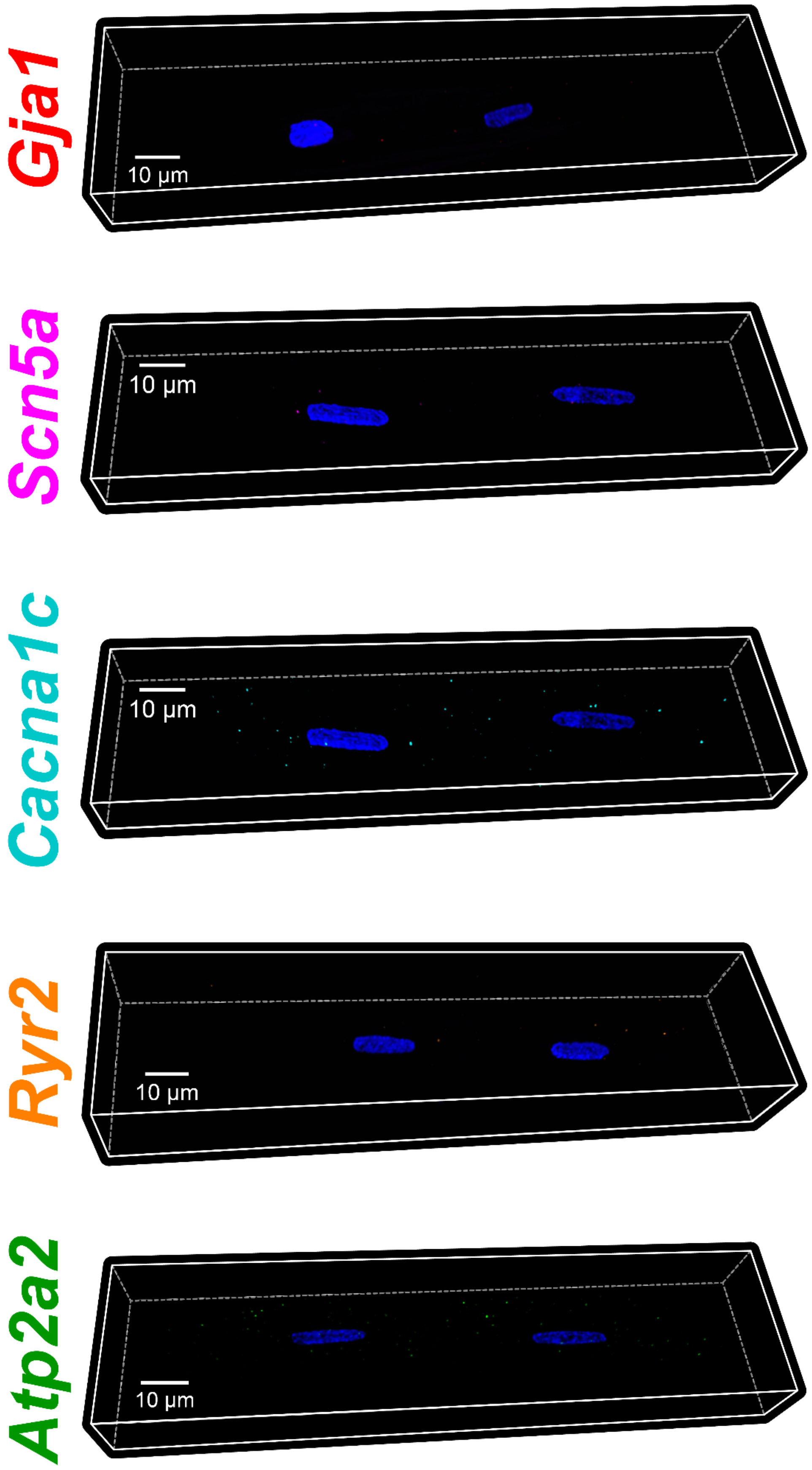

Figure S2. Representative confocal images of RNAScope signals from negative control experiments where probes designed for mouse mRNA were applied to rat cardiac myocytes.

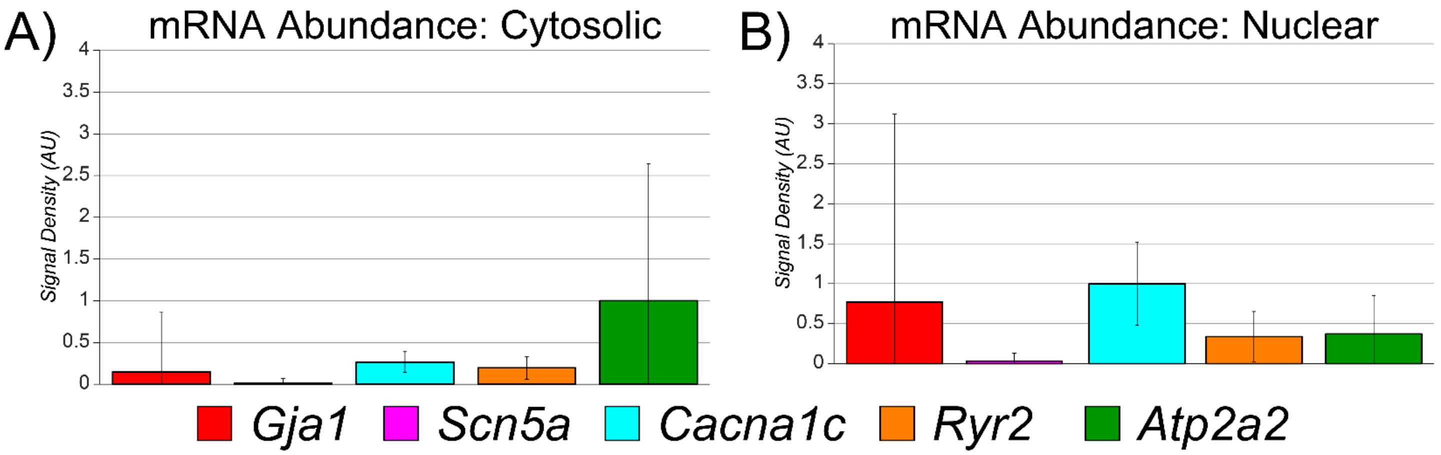

	Cacna1c	*Scn5a*	*Ryr2*	*Atp2a2*
Gja1	-	*	-	*
Cacna1c		*	-	*
Scn5a			*	*
Ryr2				*

	Cacna1c	*Scn5a*	*Ryr2*	*Atp2a2*
Gja1	-	*	-	-
Cacna1c		*	#	-
Scn5a			*	*
Ryr2				-

Figure S3. Summary plots showing volume-normalized abundance of different mRNA species within the **A)** cytosol and **B)** nucleus. Tables: Results from Bonferroni-corrected Wilcoxon's test. * $p < 0.01$, # $p < 0.05$, - p = ns. (*Gja1* [Cx43]: n = 10 cells from 3 hearts; *Scn5a* [$\mathrm{Na_V}$1.5]: n = 16 cells from 3 hearts; *Cacna1c* [$\mathrm{Ca_V}$1.2]: n = 16 cells from 3 hearts; *Ryr2* [RyR2]: n = 16 cells from 3 hearts; *Atp2a2* [Serca2a]: n = 15 cells from 3 hearts)

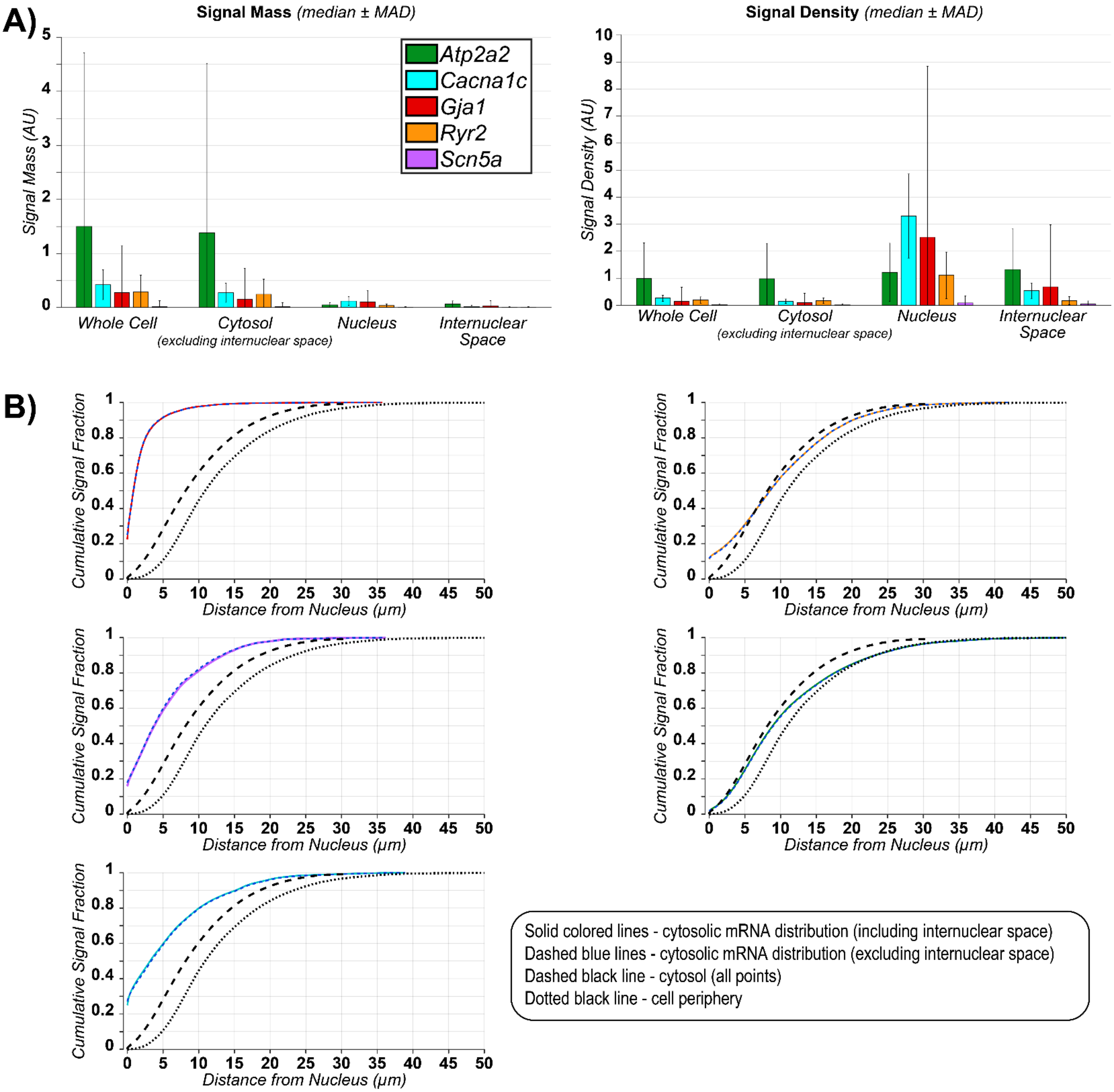

Figure S4. A) Density and mass of RNAScope signals within different cellular regions. **B)** Cumulative distribution functions of mRNAs signal vs. distance from the nucleus with standard deviations indicated by shaded regions. Solid lines show signals in the cytosol including the inter-nuclear space and dashed blue lines show signals in the cytosol excluding the inter-nuclear space. (*Gja1* [Cx43]: n = 10 cells from 3 hearts; *Scn5a* [Na_V1.5]: n = 16 cells from 3 hearts; *Cacna1c* [Ca_V1.2]: n = 16 cells from 3 hearts; *Ryr2* [RyR2]: n = 16 cells from 3 hearts; *Atp2a2* [Serca2a]: n = 15 cells from 3 hearts)

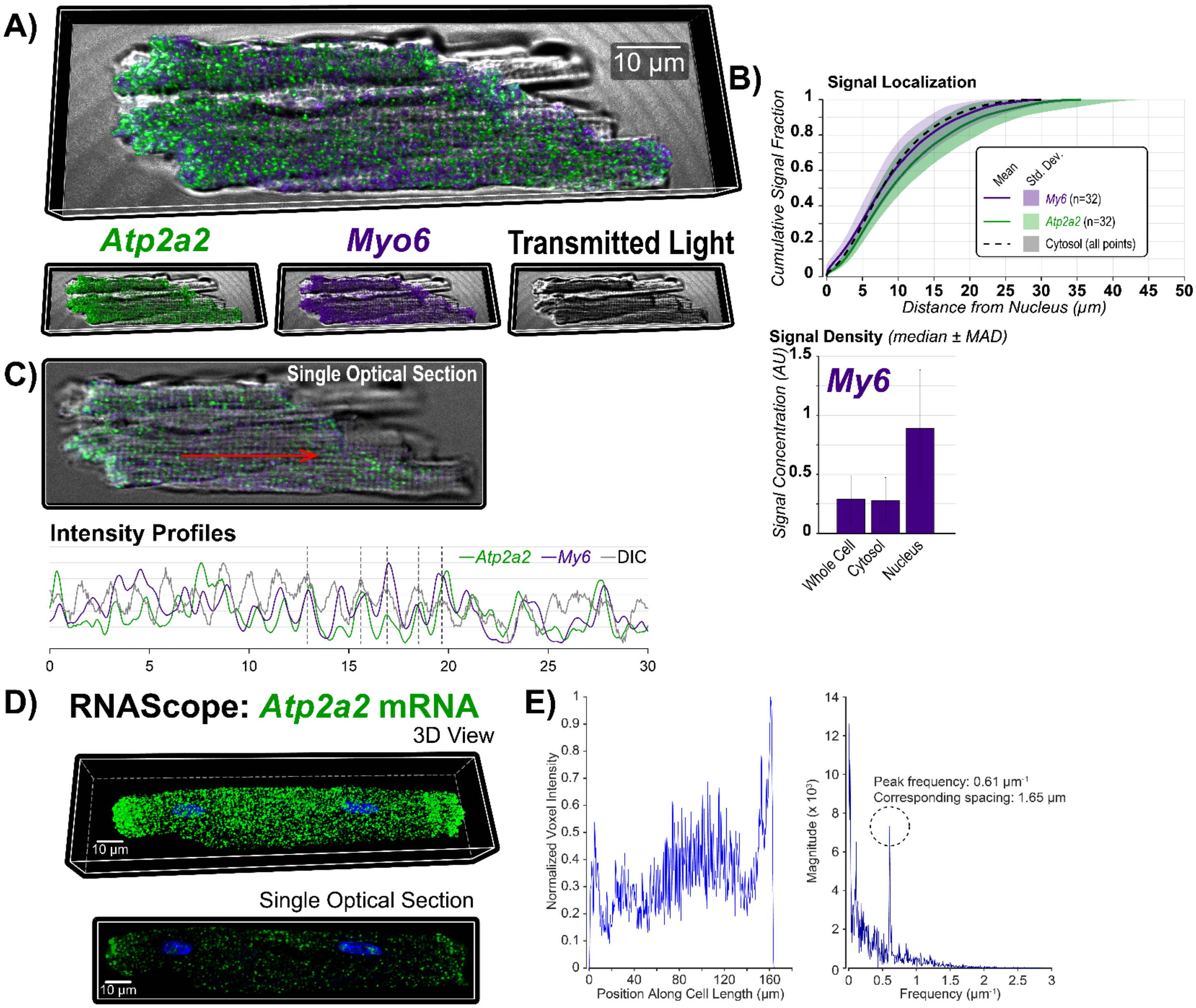

Figure S5. A) Representative 3D confocal image of an adult cardiac myocyte showing *Atp2a2* (encoding membrane protein Serca2a) and *Myh6* (encoding cytosolic protein myosin 6) mRNA. **B)** CDF showing mRNA localization relative to the nuclei (top) and *My6* mRNA abundance (bottom). Dashed black line in the localization graph shows the CDF for all voxels within the cytosolic space. *Atp2a2* mRNA abundance data are presented in Figure S3. **C)** Single optical section from the image shown in A (left) and linear intensity profile of mRNA signals along the red arrow (right). Dashed black vertical lines highlight alignment of *Atp2a2* and *My6* signals with sarcomeric bright bands (peaks) of the transmitted light image. **D)** Representative 3D confocal image (top) and single optical section (bottom) showing *Atp2a2* mRNA. **E)** Fluorescence intensity profile (left) and magnitude of the Fourier transform of fluorescence intensity (right) showing a peak at 0.61 µm^{-1}, which corresponds to 1.65 µm spacing. (*Atp2a2*: n = 15 cells from 3 hearts; *My6*: n = 32 cells from 4 hearts)

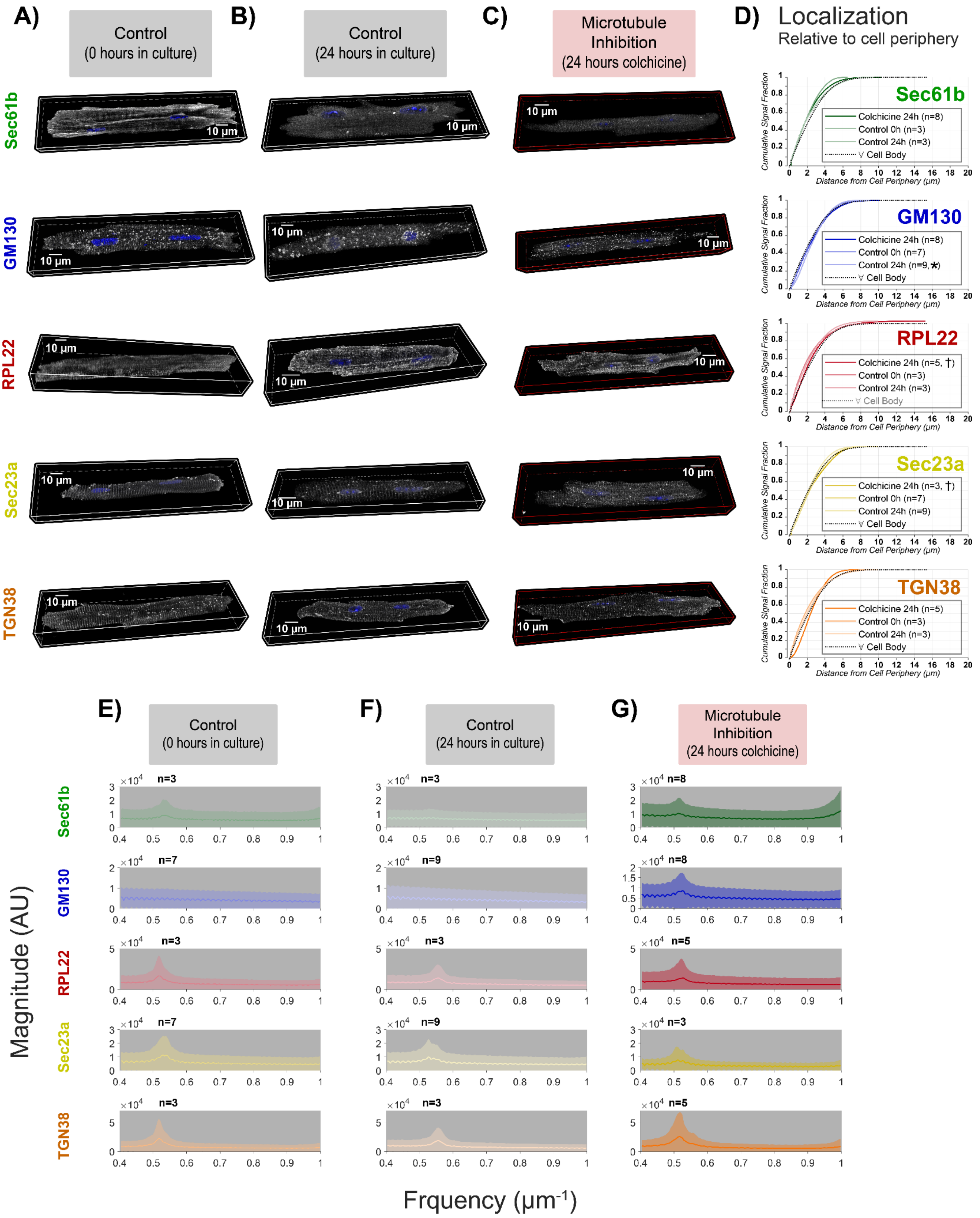

Figure S6. Representative 3D confocal images of protein transport protein Sec61b, part of the translocon complex, Cis-Golgi protein GM130, ribosomal protein Rpl22 (associated with actively translating ribosomes), protein transport protein Sec23a, component of COPII, and trans-Golgi network protein TGN38 from cardiac

myocytes that were **A)** freshly isolated (0 hours in culture) or cultured for 24 hours in the **B)** absence (control), and **C)** presence of microtubule inhibitor, colchicine. In all cases, proteins are shown in grayscale with nuclei in blue. **D)** Cumulative distribution functions showing distribution of immunosignals relative to cell periphery. (n's indicated within figure; * $p<0.05$ vs. 0 hour control, † $p<0.05$ vs. 24 hour control). Dashed black lines indicate the CDFs for all voxels within the cytosolic space relative to the cell periphery. **E-G)** Fast Fourier transform power spectra illustrating periodic distribution of Sec61b, RPL22, Sec23a and TGN38, but not GM130. In each case, the average power spectrum from all cells tested is shown as a line while the standard deviation of the power spectrum is indicated by the shaded region. Note: Representative images in 6A are repeated from Figure 2 in order to facilitate easy comparison with other conditions.

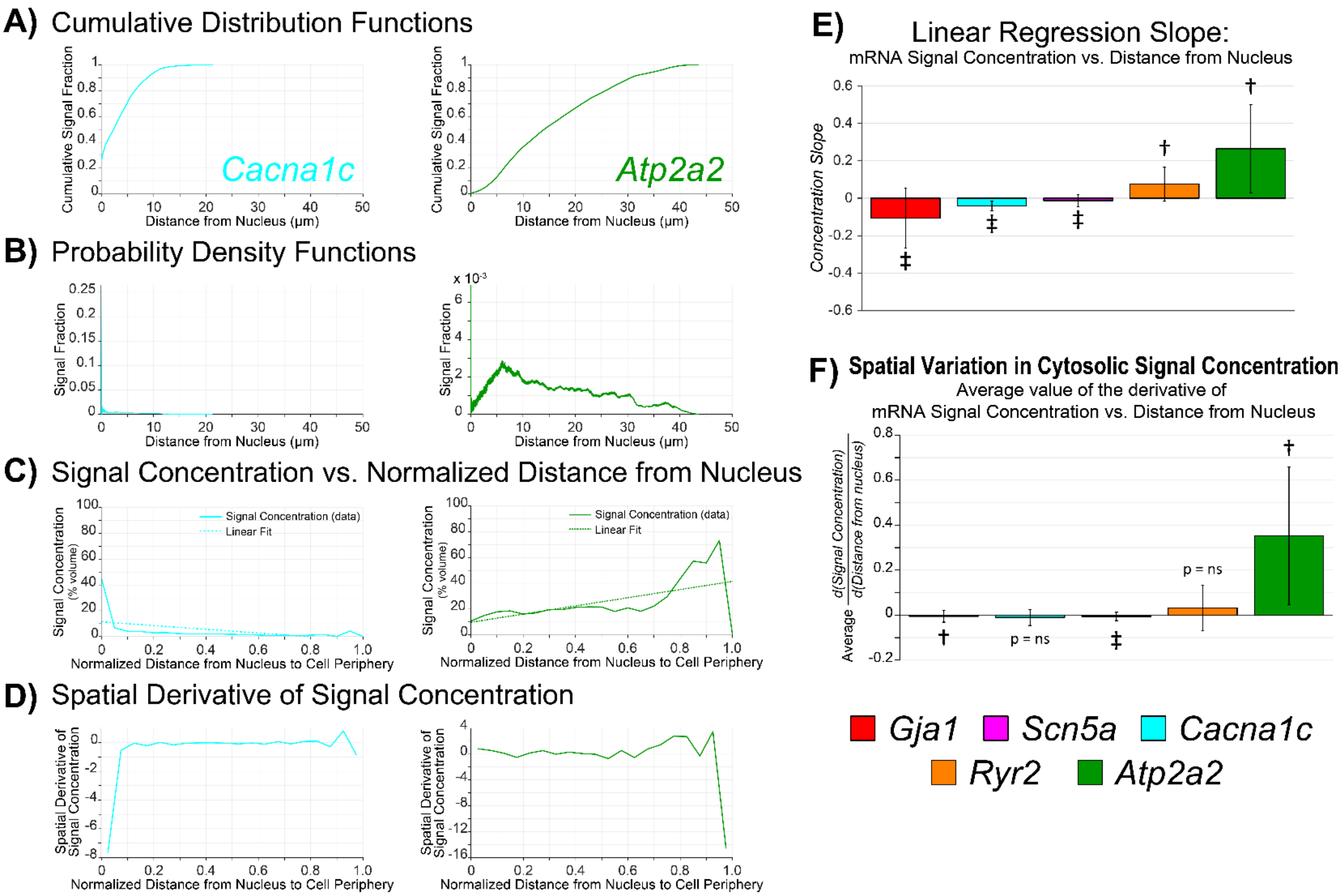

Figure S7. A) Cumulative distributions, **B)** Probability density functions, **C)** Signal concentration (% voxels occupied) vs. distance plots, and **D)** Spatial derivative of signal concentration are shown for *Cacna1c* [$Ca_V1.2$] and *Atp2a2* [Serca2a]. **E)** Slope of linear regression and **F)** Average value of the first spatial derivative of mRNA signal concentration as a function of distance from the nuclei. † $p < 0.05$, ‡ $p < 0.01$ vs. 0 from signed rank test. (*Cacna1c*: n = 16 cells from 3 hearts; *Atp2a2*: n = 15 cells from 3 hearts)

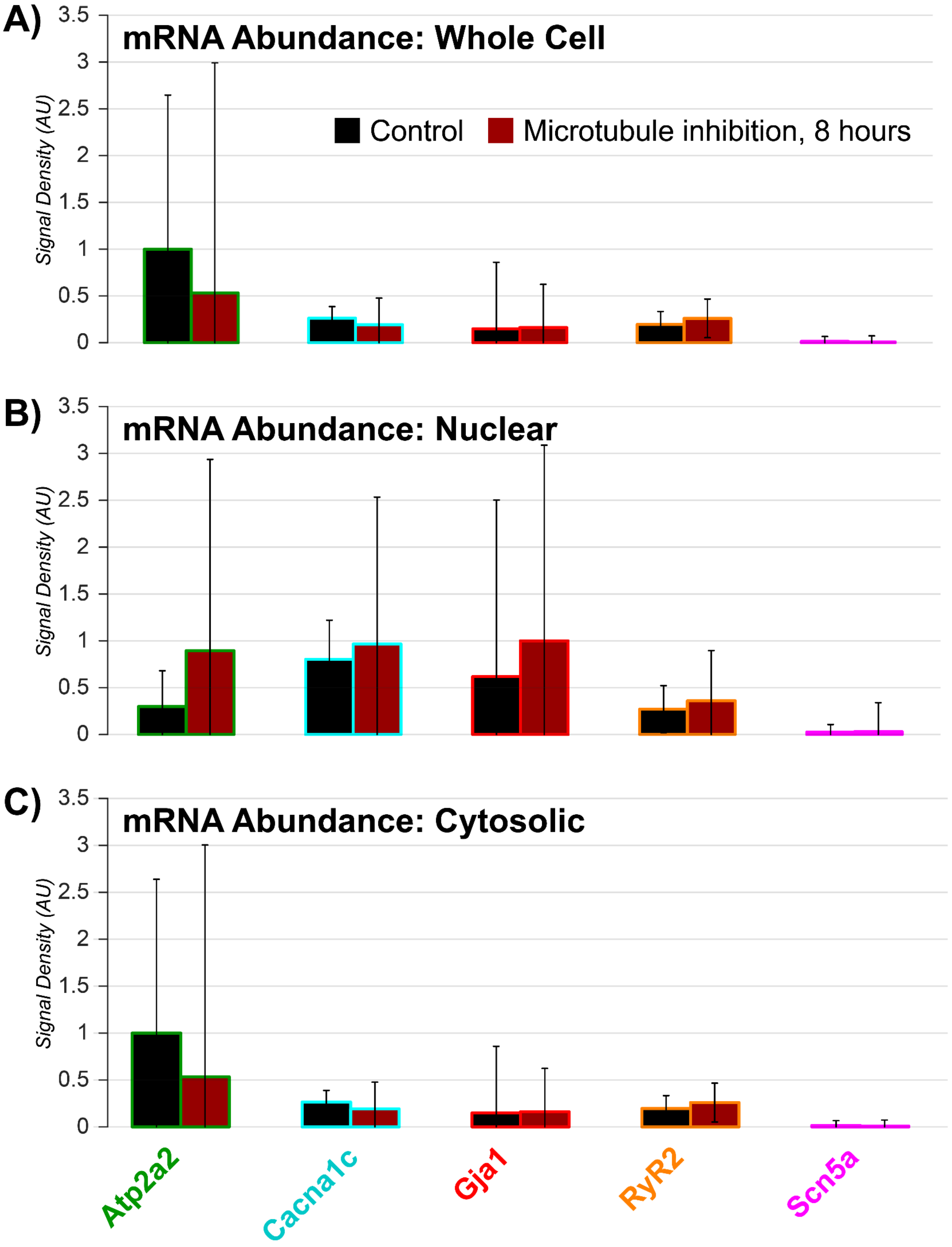
A)
mRNA Abundance: Whole Cell
Control
Microtubule inhibition, 8 hours
B)
mRNA Abundance: Nuclear
C)
mRNA Abundance: Cytosolic
Signal Density (AU)
3.5
3
2.5
2
1.5
1
0.5
0
Atp2a2
Cacna1c
Gja1
RyR2
Scn5a

Figure S8. Summary plots showing abundance of mRNA under control conditions (black) and following microtubule inhibition (red): **A)** Whole cell, **B)** Nuclear, and **C)** Cytosolic. (Control: *Gja1* [Cx43]: n = 10 cells from 3 hearts; *Scn5a* [Na_V1.5]: n = 16 cells from 3 hearts; *Cacna1c* [Ca_V1.2]: n = 16 cells from 3 hearts; *Ryr2* [RyR2]: n = 16 cells from 3 hearts; *Atp2a2* [Serca2a]: n = 15 cells from 3 hearts; Microtubule inhibition: *Gja1* [Cx43]: n = 10 cells from 3 heart; *Scn5a* [Na_V1.5]: n = 15 cells from 3 hearts; *Cacna1c* [Ca_V1.2]: n = 15 cells from 3 hearts; *Ryr2* [RyR2]: n = 15 cells from 3 hearts; *Atp2a2* [Serca2a]: n = 16 cells from 3 hearts)

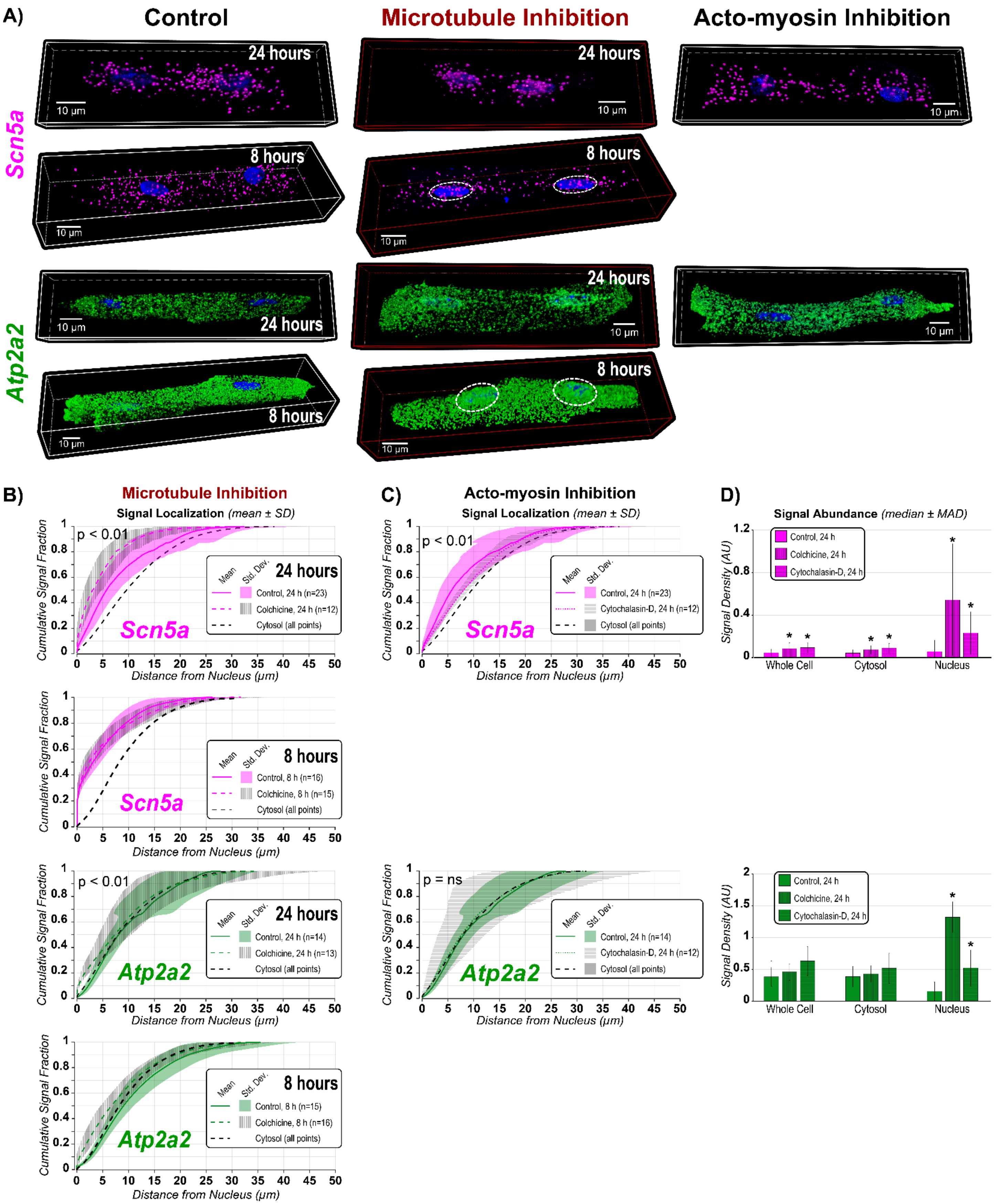

Figure S9. A) Representative 3D confocal images of *Scn5a* ($Na_V1.5$; top) and *Atp2a2* (Serca2a; bottom) mRNA from myocytes cultured for 24 hours under control conditions (left), and in the presence of microtubule inhibitor, colchicine (center), and acto-myosin inhibitor, cytochalasin-D. For ease of comparison, images obtained after 8 hours of culture under control conditions (left), and in the presence of microtubule inhibitor, colchicine (center) are repeated here from main figure 3. **B, C)** CDFs of mRNA localization relative to nuclei and **C)** mRNA

abundance in the whole cell, cytosol and nucleus. Dashed black lines in B, C show CDFs for all voxels within the cytosolic space. * $p < 0.01$ vs. control. (*Scn5a*: n = 16 cells from 3 hearts; *Atp2a2*: n = 15 cells from 3 hearts) For ease of comparison, localization CDFs obtained after 8 hours of culture under control conditions (left), and in the presence of microtubule inhibitor, colchicine (center) are repeated here from main figure 3.

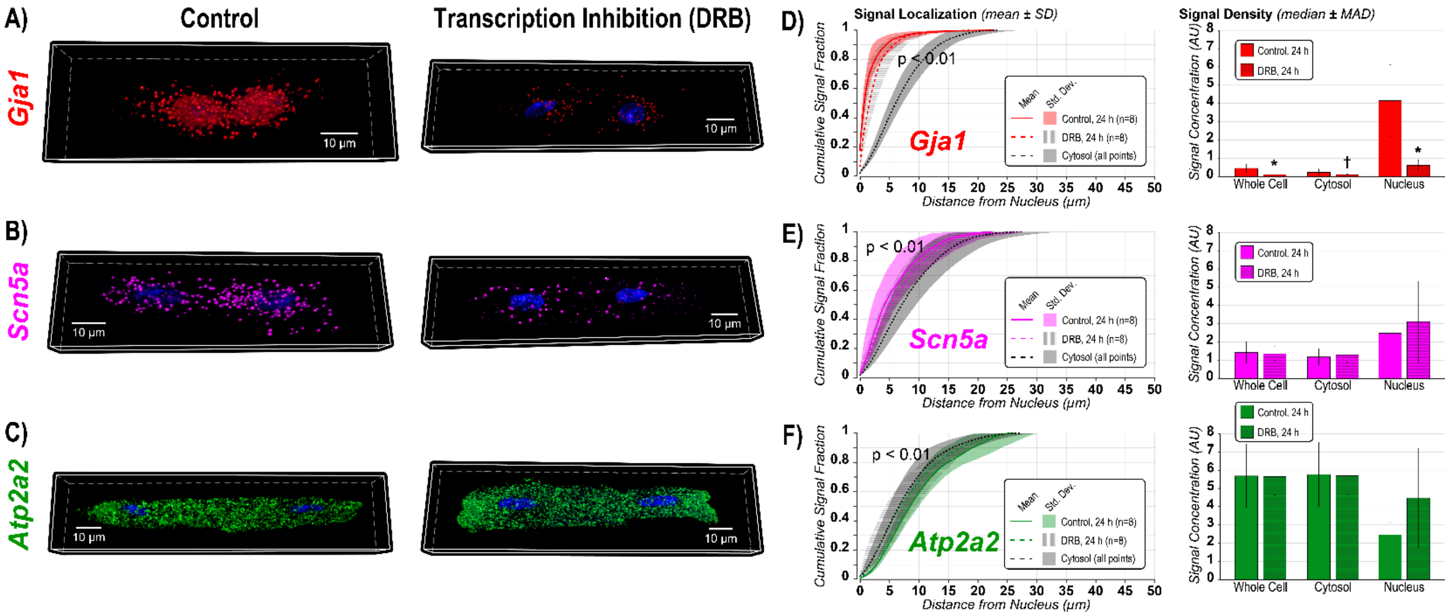

Figure S10. A-C) Representative 3D confocal images of mRNA from myocytes cultured for 24 hours in the absence (control) and presence of transcription inhibitor, DRB. **D-F)** CDFs of signal localization relative to the nucleus and bar graphs of signal density in the cytosol. * $p < 0.01$ vs. control ($n = 8$ cells per group from 1 heart)

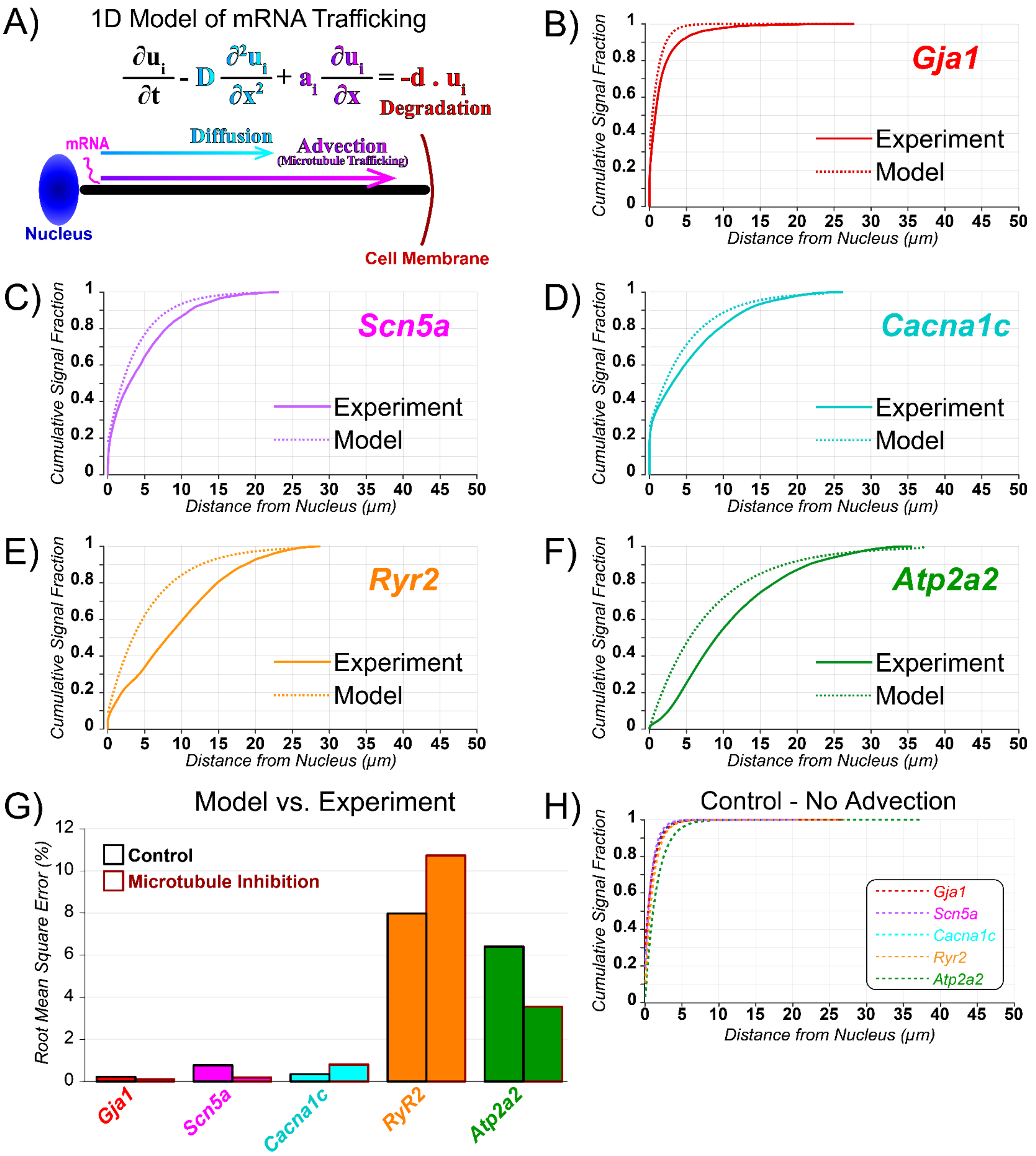

$$\frac{\partial u_i}{\partial t} - D\frac{\partial^2 u_i}{\partial x^2} + a_i\frac{\partial u_i}{\partial x} = -d \cdot u_i$$

Figure S11. A) Schematic of the 1D diffusion – advection model. **B-F)** Experimentally-derived (solid lines) and model-predicted (dashed lines) cumulative distributions of mRNA signals vs. distance from nuclei under control conditions. **G)** Root mean squared (RMS) error between model and experimental mRNA distributions under control conditions and following microtubule inhibition. **H)** Model-predicted cumulative distributions of mRNA signals vs. distance from nuclei in the absence of advection (diffusion only).

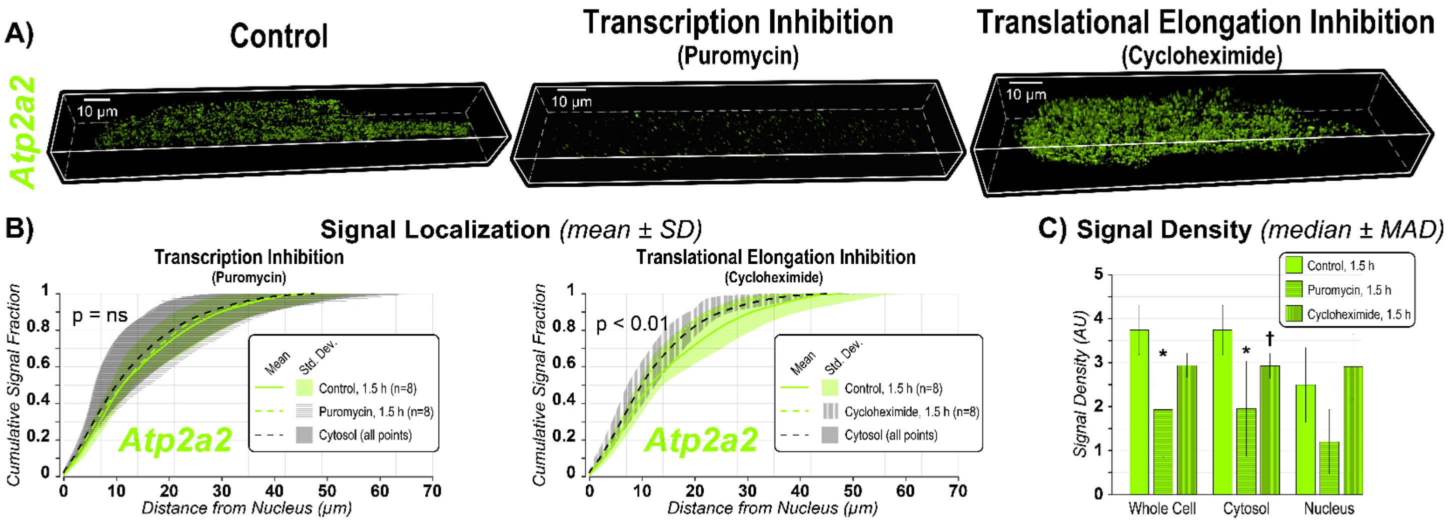

Figure S12. A) Representative 3D confocal images of ribosome-associated *Atp2a2* mRNA (encoding Serca2a; MR-PLISH) from myocytes treated for 90 minutes with vehicle (control; left), translation inhibitor, puromycin (center), and translation elongation inhibitor (which prevents mRNA-ribosome dissociation), cycloheximide (right). **B)** CDFs of ribosome-associated *Atp2a2* mRNA localization relative to nuclei and **C)** bar graph of signal abundance. * p<0.01 vs. control. (8 cells per group from 1 heart)

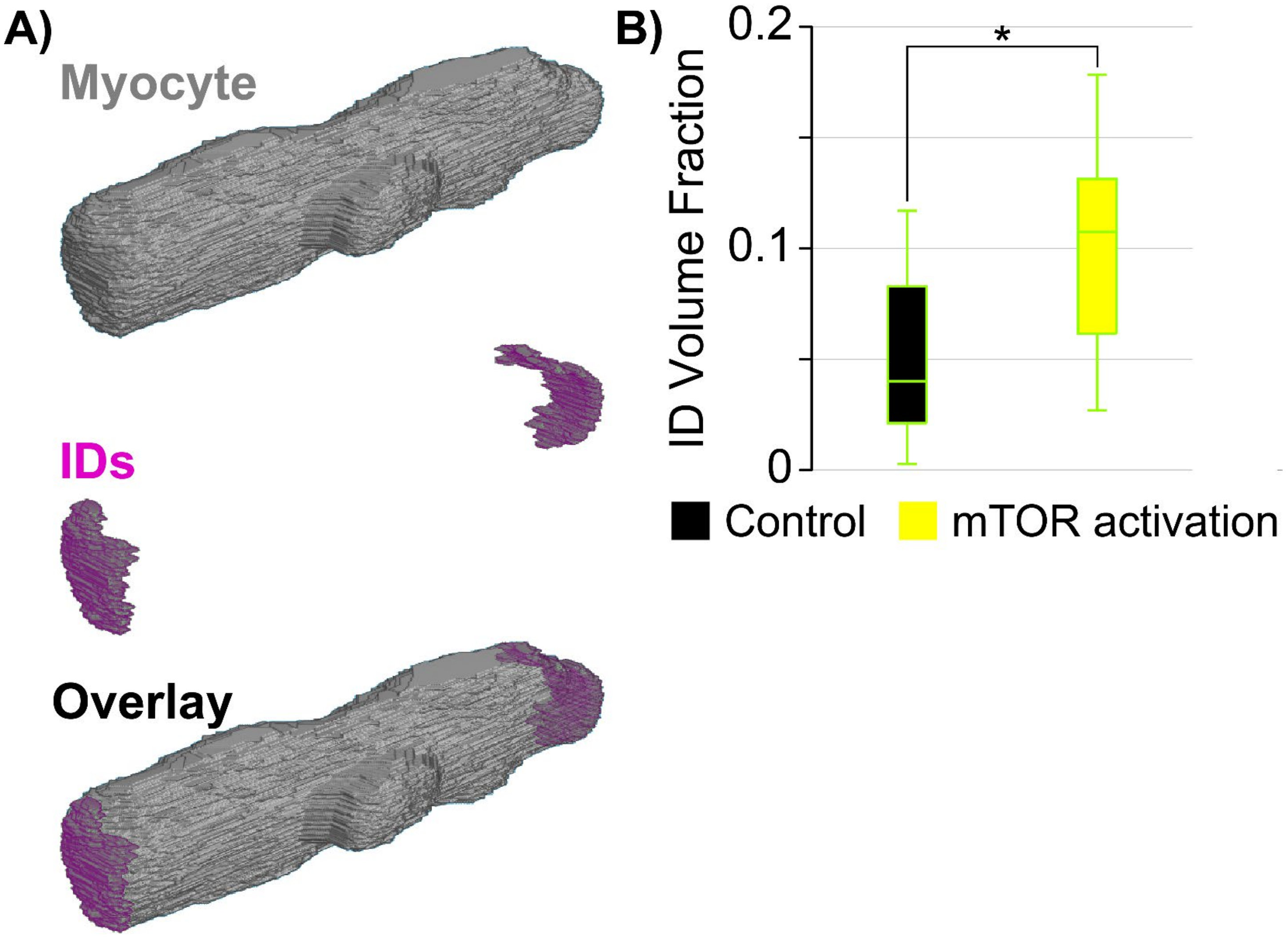

Figure S13. A) Representative 3D image showing whole cell mask (gray) of an isolated myocyte derived from a confocal image, and intercalated disk regions (pink). **B)** Volume fraction of intercalated-disk associated cellular compartments taken up by potential *Atp2a2* [Serca2a] translation sites under control conditions (black) and following mTOR activation (yellow). * Wilcoxon's test: $p < 0.01$. (n = 21 cells / group from 3 hearts)

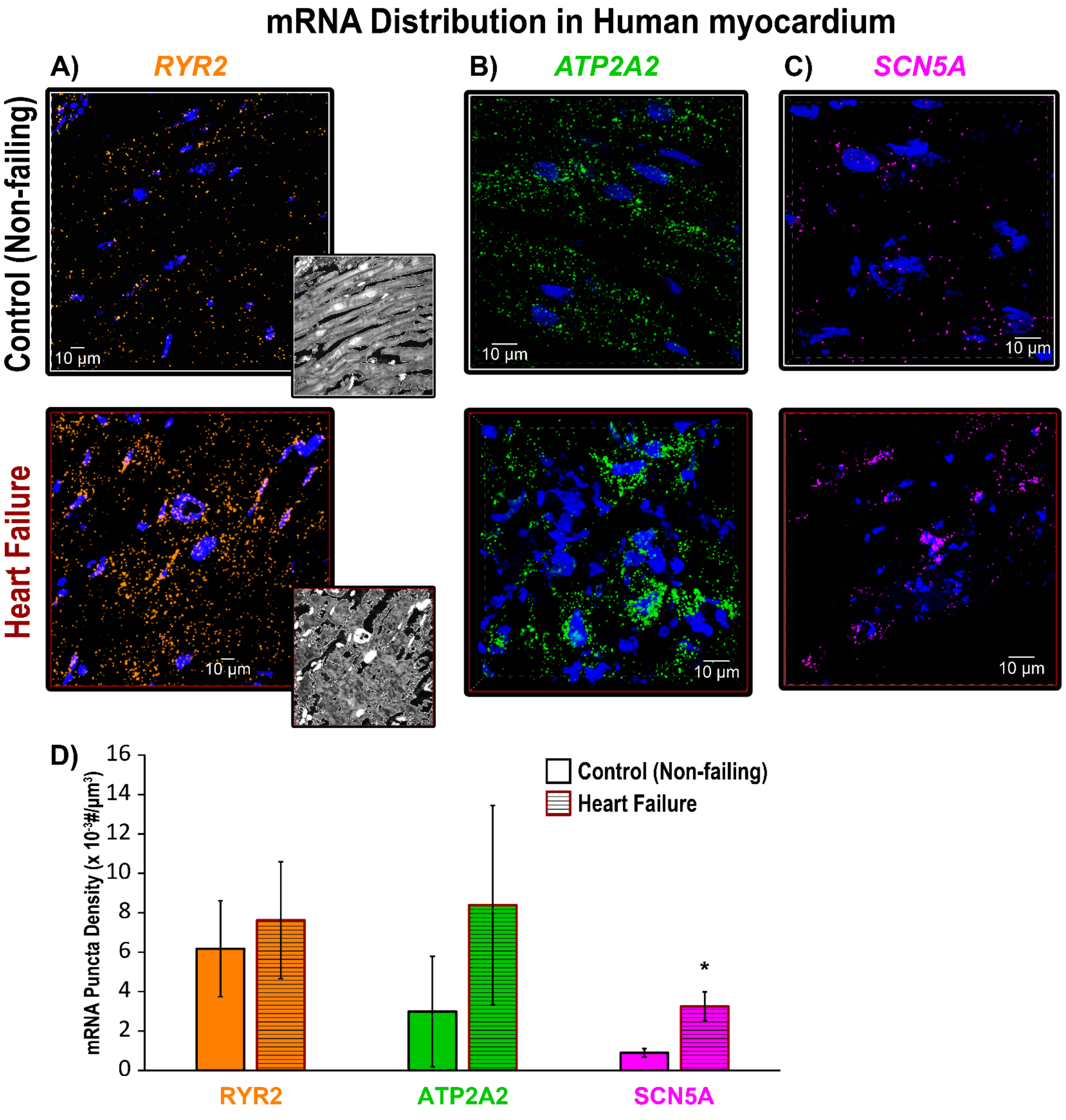

Figure S14. Representative confocal images of non-failing and failing human myocardial sections showing the presence of **A)** RYR2 (encoding RyR2), **B)** ATP2A2 (encoding Serca2A) and **C)** SCN5A (encoding $Na_V1.5$) mRNA throughout the cells. Inset panels in 6A show the outlines of the tissue segmented from the nuclear label to aid with interpretation. **D)** Density of mRNA puncta normalized to tissue volume. * Student's t-test: $p < 0.05$. (n = 15 images from 3 hearts per group)

REFERENCES

1. Ackers-Johnson M, Li PY, Holmes AP, O'Brien SM, Pavlovic D, Foo RS (2016) A Simplified, Langendorff-Free Method for Concomitant Isolation of Viable Cardiac Myocytes and Nonmyocytes From the Adult Mouse Heart. Circulation research 119:909-920 doi:10.1161/CIRCRESAHA.116.309202
2. Lin SJ, Chang KP, Hsu CW, Chi LM, Chien KY, Liang Y, Tsai MH, Lin YT, Yu JS (2013) Low-molecular-mass secretome profiling identifies C-C motif chemokine 5 as a potential plasma biomarker and therapeutic target for nasopharyngeal carcinoma. J Proteomics 94:186-201 doi:10.1016/j.jprot.2013.09.013
3. Mezache L, Struckman HL, Greer-Short A, Baine S, Gyorke S, Radwanski PB, Hund TJ, Veeraraghavan R (2020) Vascular endothelial growth factor promotes atrial arrhythmias by inducing acute intercalated disk remodeling. Sci Rep 10:20463 doi:10.1038/s41598-020-77562-5
4. Nagendran M, Riordan DP, Harbury PB, Desai TJ (2018) Automated cell-type classification in intact tissues by single-cell molecular profiling. Elife 7 doi:10.7554/eLife.30510
5. Ralston E, Lu Z, Ploug T (1999) The organization of the Golgi complex and microtubules in skeletal muscle is fiber type-dependent. J Neurosci 19:10694-10705
6. Struckman HL, Baine S, Thomas J, Mezache L, Mykytyn K, Gyorke S, Radwanski PB, Veeraraghavan R (2020) Super-Resolution Imaging Using a Novel High-Fidelity Antibody Reveals Close Association of the Neuronal Sodium Channel NaV1.6 with Ryanodine Receptors in Cardiac Muscle. Microsc Microanal:1-9 doi:10.1017/S1431927619015289
7. Watanabe T, Sakai Y, Koga D, Bochimoto H, Hira Y, Hosaka M, Ushiki T (2012) A unique ball-shaped Golgi apparatus in the rat pituitary gonadotrope: its functional implications in relation to the arrangement of the microtubule network. The journal of histochemistry and cytochemistry : official journal of the Histochemistry Society 60:588-602 doi:10.1369/0022155412448791
8. Zacharogianni M, Aguilera-Gomez A, Veenendaal T, Smout J, Rabouille C (2014) A stress assembly that confers cell viability by preserving ERES components during amino-acid starvation. Elife 3 doi:10.7554/eLife.04132
9. Zhang R, Miner JJ, Gorman MJ, Rausch K, Ramage H, White JP, Zuiani A, Zhang P, Fernandez E, Zhang Q, Dowd KA, Pierson TC, Cherry S, Diamond MS (2016) A CRISPR screen defines a signal peptide processing pathway required by flaviviruses. Nature 535:164-168 doi:10.1038/nature18625
